# Unraveling the Mechanism of Xiaochaihu Granules in Alleviating Yeast-Induced Fever Based on Network Analysis and Experimental Validation

**DOI:** 10.3390/ph17040475

**Published:** 2024-04-08

**Authors:** Xiuli Chen, Hao Wu, Peibo Li, Wei Peng, Yonggang Wang, Xiaoli Zhang, Ao Zhang, Jinliang Li, Fenzhao Meng, Weiyue Wang, Weiwei Su

**Affiliations:** Guangdong Engineering & Technology Research Center for Quality and Efficacy Reevaluation of Post-Market Traditional Chinese Medicine, Guangdong Provincial Key Laboratory of Plant Resources, State Key Laboratory of Biocontrol, School of Life Sciences, Sun Yat-sen University, Guangzhou 510275, China

**Keywords:** Xiaochaihu granules, fever, network analysis, experimental validation, anti-inflammatory

## Abstract

Xiaochaihu granules (XCHG) are extensively used to treat fever. Nevertheless, the underlying mechanism remains elusive. This study aimed to explore the potential of XCHG in mitigating yeast-induced fever and the underlying metabolic pathways. The chemical composition of XCHG was ascertained using ultra-fast liquid chromatography/quadrupole-time-of-flight tandem mass spectrometry (UFLC-Q-TOF-MS/MS), followed by integrated network analysis to predict potential targets. We then conducted experimental validation using pharmacological assays and metabolomics analysis in a yeast-induced mouse fever model. The study identified 133 compounds in XCHG, resulting in the development of a comprehensive network of herb–compound–biological functional modules. Subsequently, molecular dynamic (MD) simulations confirmed the stability of the complexes, including γ-aminobutyric acid B receptor 2 (GABBR2)–saikosaponin C, prostaglandin endoperoxide synthases (PTGS2)–lobetyolin, and NF-κB inhibitor IκBα (NFKBIA)–glycyrrhizic acid. Animal experiments demonstrated that XCHG reduced yeast-induced elevation in NFKBIA’s downstream regulators [interleukin (IL)-1β and IL-8], inhibited PTGS2 activity, and consequently decreased prostaglandin E2 (PGE2) levels. XCHG also downregulated the levels of 5-hydroxytryptamine (5-HT), γ-aminobutyric acid (GABA), corticotropin releasing hormone (CRH), and adrenocorticotrophin (ACTH). These corroborated the network analysis results indicating XCHG’s effectiveness against fever in targeting NFKBIA, PTGS2, and GABBR2. The hypothalamus metabolomics analysis identified 14 distinct metabolites as potential antipyretic biomarkers of XCHG. In conclusion, our findings suggest that XCHG alleviates yeast-induced fever by regulating inflammation/immune responses, neuromodulation, and metabolism modules, providing a scientific basis for the anti-inflammatory and antipyretic properties of XCHG.

## 1. Introduction

Fever, a common clinical manifestation in various diseases, is characterized by a regulated increase in the body’s temperature set point in response to pyrogenic stimuli [1,2]. This physiological response is a complex defensive mechanism against infections. Acetaminophen (APAP) is commonly used to alleviate fever [3]. However, APAP often poses a risk of recurrence [4] because it fails to address the underlying cause of the fever [5]. Additionally, it causes adverse effects, such as skin rash, urticaria, and thrombocytopenia [6,7]. Traditional Chinese medicine (TCM), offering a comprehensive treatment strategy that addresses the disease’s symptoms and underlying causes, has been employed in fever treatment for centuries [8,9,10]. TCM leverages multi-component approaches to target multiple nodes, thereby regulating the disease network and enhancing synergistic effects [11]. Xiaochaihu granules (XCHG) are widely used in clinical practice for treating fever-related conditions and ameliorating symptoms, such as anepithymia, nausea, and pain [12]. Notably, during the coronavirus disease 2019 (COVID-19) pandemic, XCHG demonstrated significant potential in alleviating fever, a common symptom of the disease [13]. Importantly, XCHG has minimal toxic effects and is safe for use in pregnant women and children [14,15]. Of course, if XCHG is abused without diagnosis and treatment, it can also cause toxic effects. For example, an incident of pneumonitis caused by XCHG in Japan has been reported [16]. Apart from that, few reports of side effects caused by XCHG have been published.

XCHG is derived from the classic formula “Xiaochaihu Decoction”, documented in the ancient Chinese medical text Shanghanlun (Treatise on Febrile Diseases) by Zhang Zhongjing. It is composed of seven medicinal plant components: the dried root of *Bupleurum chinense* DC. [Apiaceae] (Chaihu, CH), 31.0%, the dried root of *Scutellaria baicalensis* Georgi [Lamiaceae] (Huangqin, HQ), 11.5%, the ginger-processed *Pinellia ternata* (Thunb.) Makino [Araceae] (Jiangbanxia, JBX), 11.5%, the dried root of *Codonopsis pilosula* (Franch.) Nannf. [Campanulaceae] (Dangsheng, DS), 11.5%, the fresh root of *Zingiber officinale* Roscoe [Zingiberaceae] (Shengjiang, SJ), 11.5%, the dried root of *Glycyrrhiza uralensis* Fisch. ex DC. [Fabaceae] (Gancao, GC), 11.5%, and the dried ripe fruit of *Ziziphus jujuba* Mill. [Rhamnaceae] (Dazao, DZ), 11.5% [17]. The pharmacognostic potential and chemical composition of the seven plants included in this formula are shown in Table 1. Despite limited reports on the antipyretic mechanism of XCHG, it has been demonstrated to relieve fever in the treatment of COVID-19 by interacting with 37 human coronavirus-associated proteins, involving the interleukin-6 (IL-6)/signal transducer and activator of transcription 3 (STAT3) pro-inflammatory signaling pathway [12]. When combined with oseltamivir, XCHG rapidly relieved fever, cough, and other symptoms in children [15]. Sun et al. (2003) proved that XCHG exerts a significant antipyretic effect on lipopolysaccharide-induced fever in rats [18]. Previous studies have reported that XCHG relieves fever and improves viral pneumonia by inhibiting pro-inflammatory cytokines (IL-1β, IL-6, and tumor necrosis factor [TNF]-α) [19,20]. These findings highlight the potential of XCHG as an effective antipyretic treatment strategy. However, further research is needed to fully understand its mechanisms.

XCHG contains various chemicals with complex and diverse interactions. Although Bi et al. (2020) identified 66 chemical components of XCHG using ultra-high-performance liquid chromatography–tandem mass spectrometry (UHPLC-MS), it appears insufficiently comprehensive [19]. Their study solely relied on database searches and fragment analysis without employing chemical reference standards for confirmation or conducting quantitative analysis of the components. Therefore, it is necessary to conduct a more comprehensive and precise analysis of the chemical composition of XCHG to better understand the material basis of its medicinal effects.

With the rapid advancement of bioinformatics and computational biology, the integration of network analysis, molecular docking, and molecular dynamic (MD) simulation has emerged as a novel interdisciplinary field for the comprehensive study of TCM mechanisms [36,37]. Researchers have successfully employed molecular docking and MD simulation methods to screen and identify 8 out of 318 phytochemicals for the inhibition of *Xanthomonas oryzae pv. Oryzae* by targeting peptide deformylase [38]. Integrated network analysis, MD simulation, and experimental verification demonstrated that kaempferol, luteolin, and baicalein in Fufang Zhenzhu Tiaozhi capsules can significantly inhibit the activity of dipeptidyl peptidase 4 and cyclooxygenase (COX)-2 in glucose and lipid metabolism disorders [36]. Biomarkers in target tissue metabolomics serve as the pharmacodynamic surrogate indices to elucidate the mechanisms underlying the therapeutic effects of TCM. A study integrating UPLC-MS/MS, hypothalamus metabolomics, and network pharmacology analysis identified active components and mechanisms contributing to the antipyretic effects of Qingkaikling injection [39]. These findings provide valuable insights for studying the antipyretic mechanism of XCHG.

**Figure 1:**
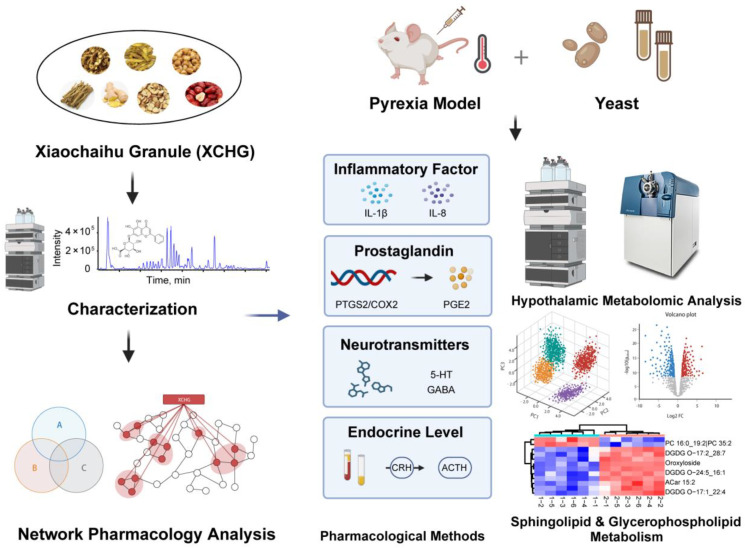
Schematic diagram of the research process.

The current study employs a combination of ultra-fast liquid chromatography/quadrupole-time-of-flight tandem mass spectrometry (UFLC-Q-TOF-MS/MS), network analysis, molecular docking, and MD simulation to determine the chemical composition of XCHG and predict its potential targets. Following this, we employed a yeast-induced fever mouse model to validate the antipyretic pathways of XCHG using both pharmacological and metabolomic approaches. The detailed methodology is illustrated in Figure 1. This comprehensive approach allows us to explore the antipyretic properties of XCHG systematically and rigorously.

## 2. Results

### 2.1. Chemical Composition of XCHG

**Figure 2:**
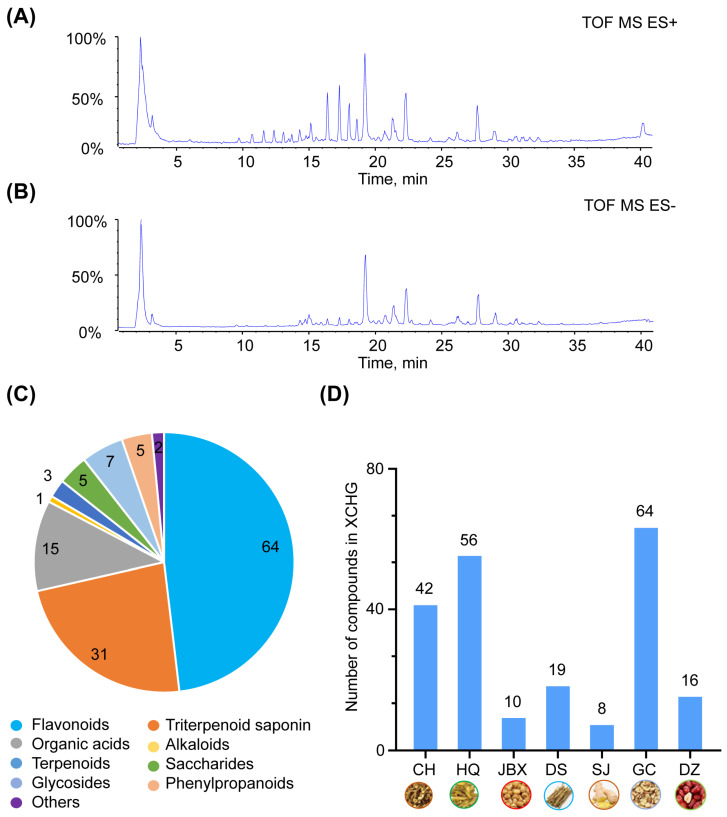
Identification of compounds in XCHG. The basic peak chromatograms (BPCs) of XCHG in the positive ion mode (**A**) and in the negative ion mode (**B**). (**C**) Compounds classification in XCHG based on the chemical structure. (**D**) The number of compounds for each herb.

The representative basic peak chromatograms (BPCs) of XCHG in both positive and negative modes are depicted in Figure 2A,B. In total, 133 components were identified, encompassing 64 flavonoids, 31 triterpenoid saponins, 15 organic acids, 1 alkaloid, 3 terpenoids, 5 saccharides, 7 glycosides, 5 phenylpropanoids, and 2 others (Figure 2C). Among these, 34 compounds were validated by comparing the formula, retention time (tR), adducts, fragment ions, and the relative intensities of the fragment *m*/*z* between the sample and reference standards (Table S1). Figure 2D presents the number of ingredients per herb (CH 42, HQ 56, JBX 10, DS 19, SJ 8, GC 64, and DZ 16). Detailed information about the formula, retention time (t_R_), adducts, fragment ions, identification, structure type, and source of XCHG chemical components are presented in Table S2.

A total of nine compounds, including baicalin, wogonin, wogonoside, baicalein, quercetin, saikosaponin B1, saikosaponin B2, glycyrrhizic acid, and liquiritin, were quantified using calibration curves of reference standards (Table S3). The calibration curves were established and we obtained a linear regression equation (R^2^ > 0.99) to calculate the analyte concentration on the basis of the peak area ratio of relevant reference standards, whereas for the concentration beyond the range of calibration curves, samples were diluted based on the practical situation. Notably, baicalin emerged as the most abundant compound (3.25 mg/g), consistent with specifications of *the Pharmacopoeia of the People’s Republic of China* (2020), which stipulates that each bag (containing 10 g of XCHG) must contain no less than 20 mg of baicalin.

### 2.2. XCHG Affects the Biological Functional Modules Related to Fever

**Figure 3:**
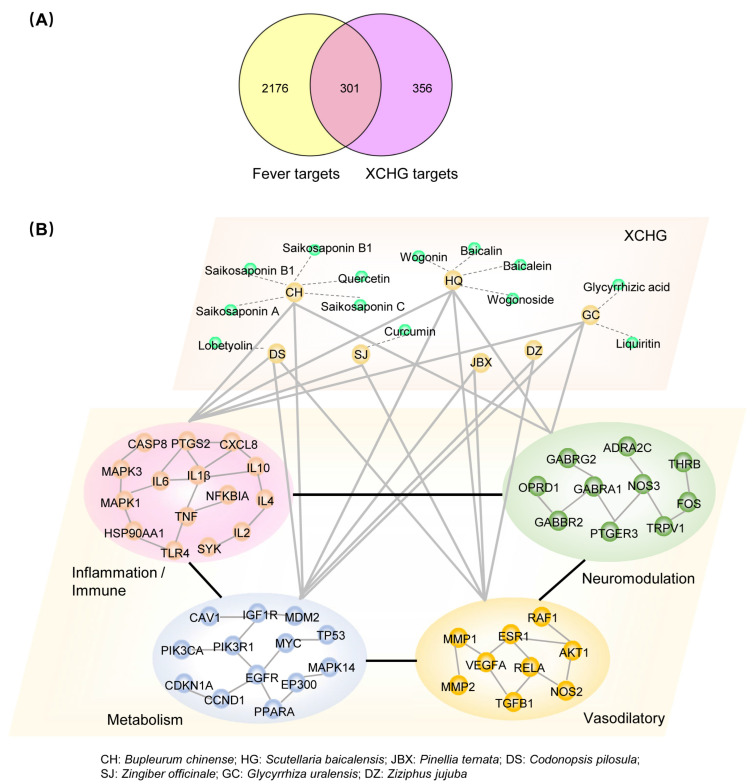
Network pharmacological analysis. (**A**) Targets’ Venn diagram of XCHG against fever. (**B**) Herb–compound–biological functional module network of XCHG. Light gray lines connecting herb to biological functional module show the herb’s predicted function. Black lines between different modules mean that the genes in different modules exhibited overlap. The genes in each biological functional module were analyzed according to the PPI network.

Based on the oral bioavailability (OB) ≥ 30%, drug-likeness (DL) ≥ 0.18, absorption, distribution, metabolism, and excretion (ADME) attribute values, and the reported active compounds, we rigorously filtered and identified 30 active ingredients of XCHG (Table S4), along with their 657 associated targets. At the same time, we conducted an extensive search for fever-related targets, amalgamated the findings, and eliminated any duplicates to obtain a comprehensive list of 2477 fever-related targets. By intersecting the XCHG targets with fever targets, we identified 301 overlapping targets responsible for the antipyretic effects of XCHG (Figure 3A).

Utilizing the DAVID database to analyze these 301 targets, we identified significantly enriched GO/KEGG terms predominantly clustered within the modules of inflammation/immune, neuromodulation, metabolism, and vasodilatory (Table S5). We successfully constructed a herb–compound–biological functional module network, which visually illustrates the interplay between herbs, compounds, and biological functional modules (Figure 3B). This contributes to a deeper understanding of the combinatorial pattern of XCHG treatment for fever from the perspective of biomolecular networks. For example, CH, the predominant plant component in XCHG and with the major bioactive compounds known as saikosaponins (SSs, including SSB1, SSB2, SSC, and SSA), has been demonstrated to regulate inflammation, immune response, and neuromodulation modules by inhibiting cytokine expression (IL-1β, PGE2, and IL-10) and reducing monoamine neurotransmitter concentrations (5-HT, dopamine, and noradrenaline) [40]. HQ is recognized for its ability to alleviate respiratory tract pathology symptoms [41], prevent apoptosis in neuron cells, and control the inflammation/immune responses, neuromodulation, and vasodilation modules [42]. SJ has been shown to suppress gastric contraction, reduce blood pressure, improve intestinal transportation, and regulate inflammation/immune responses, neuromodulation, metabolism, and vasodilation modules [43,44]. GC is used for various types of coughs and asthma induced by cold or heat. It regulates inflammation/immune responses, neuromodulation, and metabolism modules [45].

### 2.3. Molecular Docking of the Active Compounds and Core Targets

**Figure 4:**
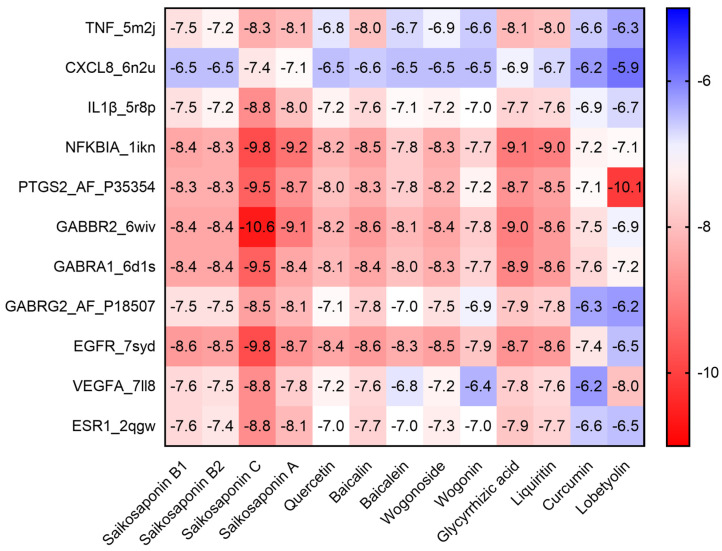
The main active compound–core target binding energies (kcal/mol) heatmap.

Molecular docking studies were conducted to explore the interactions between the main active ingredients of XCHG and their potential targets. In general, the lower the energy, the greater the possibility of receptor–ligand interaction [46,47]. The respective binding energies are displayed in Figure 4. Obviously, binding energies of most of docking complexes were below −5 kcal/mol. These results predicted the potential molecular interactions between the active compounds and the 11 core targets [TNF, CXCL8 (IL-8), IL-1β, NFKBIA, PTGS2, GABBR2, GABRA1, GABRG2, epidermal growth factor receptor (EGFR), vascular endothelial growth factor A (VEGFA), and estrogen receptor 1 (ESR1)], supporting their antipyretic effects. Many of these interactions, including baicalin–GABBR [48], saikosaponin A–GABBR [49], saikosaponin A–TNF, saikosaponin C–TNF [50], saikosaponin C–VEGFA, saikosaponin A–VEGFA [51], and glycyrrhizic acid–NFKBIA [52], have been experimentally described in the literature. Compared to other compounds, saikosaponin C had the lowest binding energies towards GABBR2, GABRA1, NFKBIA, and EGFR. Meanwhile, PTGS2–lobetyolin (−10.1 kcal/mol) showed the lowest binding energy among those of other compounds docked to PTGS2. Baicalin, the only compound for content determination of XCHG in the *Chinese Pharmacopoeia*, showed the lowest binding energy to GABBR2 during the docking process with the 11 core targets. Glycyrrhizic acid (2.05 mg/g of XCHG) had the lowest binding energy to NFKBIA.

**Figure 5:**
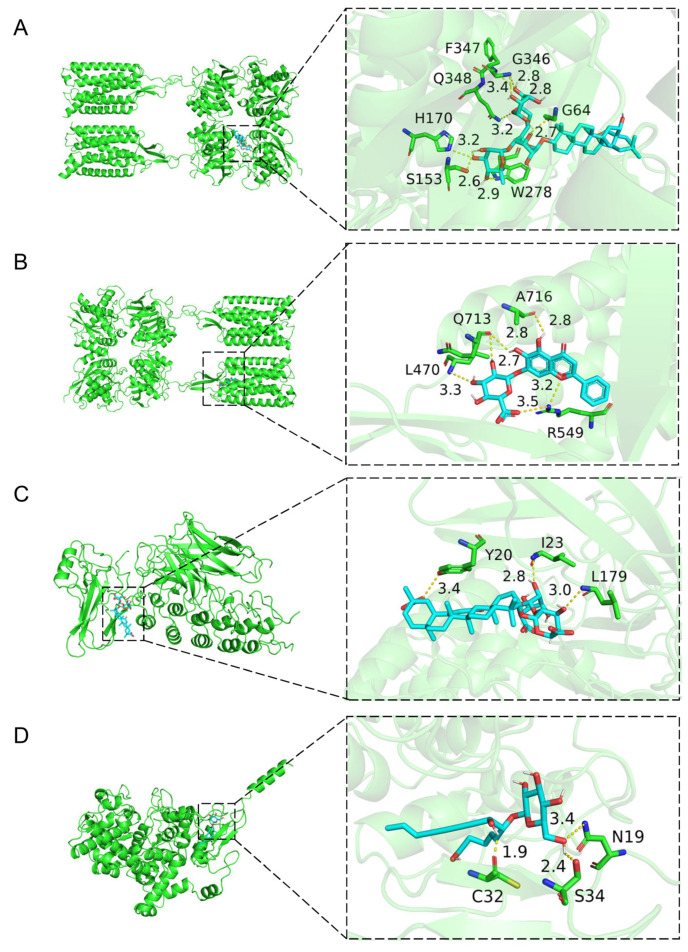
Three-dimensional mapping of compound–target interactions for GABBR2–saikosaponin C (**A**), GABBR2–baicalin (**B**), NFKBIA–glycyrrhizic acid (**C**), and PTGS2–lobetyolin (**D**).

To provide a visual representation of these interactions, three-dimensional diagrams were generated using the PyMOL 2.6.0a0 open-source software (Figure 5A–D). Saikosaponin C, a type of glycosylated epoxy-ether oleanane, is a member of the triterpenoid saponin family [40]. It carries two glucosyl and a rhamnosyl group, facilitating its binding to amino acid residues. Notably, saikosaponin C formed eight hydrogen bonds (Hbonds) with the G64, S153, H170, W278, G346, F347, and Q348 residues of GABBR2. Baicalin, a flavone glucuronide [53], formed six Hbonds with the L470, R549, Q713, and A716 residues of GABBR2. Glycyrrhizinic acid is also a triterpenoid saponin and a glucosiduronide derivative [54]. It formed three Hbonds with the Y20, I23, and L179 residues of NFKBIA. Lobetyolin, a polyacetylene glycoside [55], formed three Hbonds with the N19, C32, and S34 residues of PTGS2.

### 2.4. MD Simulations

**Figure 6:**
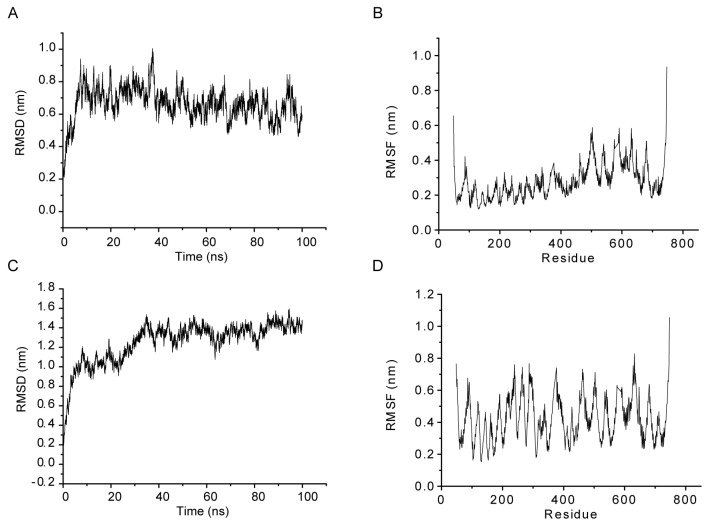

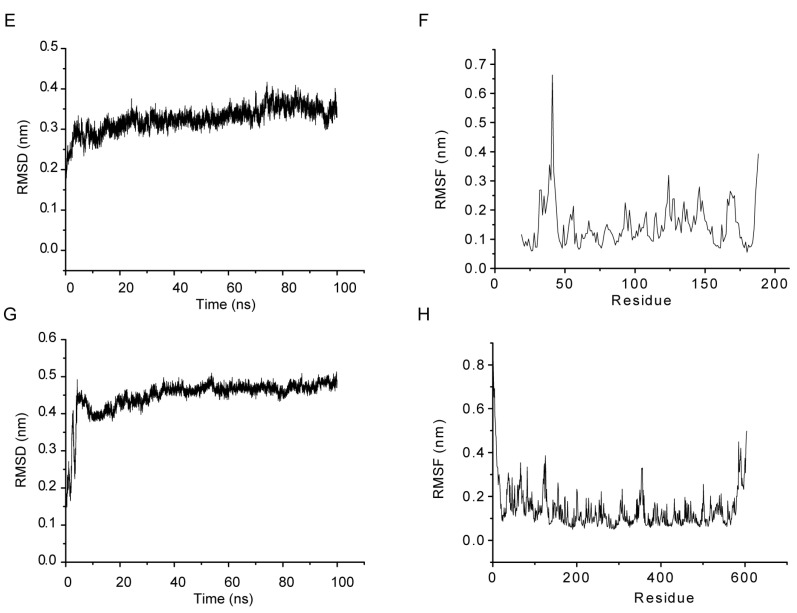
Root mean square deviations (RMSD) and root mean square fluctuations (RMSF) plot during 100 ns of MD simulations for indicated complexes: GABBR2–saikosaponin C (**A**,**B**), GABBR2–baicalin (**C**,**D**), NFKBIA–glycyrrhizic acid (**E**,**F**), and PTGS2–lobetyolin (**G**,**H**), respectively.

To further verify the stability of the main active compound–core target complexes, a 100 ns MD simulation was conducted, including GABBR2–saikosaponin C, PTGS2–lobetyolin, and NFKBIA–glycyrrhizic acid, which had high scores. Although the binding score of baicalin with each target was not high, we conducted MD simulations on the GABBR2–baicalin complex, considering that baicalin is the most abundant compound in XCHG and an important indicator for evaluating its quality. GABBR2 has been implicated in the treatment of several neurological conditions, including pain, anxiety, and spasticity [56,57]. Based on the MD analysis, root mean square deviation (RMSD) and root mean square fluctuation (RMSF) were computed to determine the stability and flexibility of the complexes during the simulations. As illustrated in Figure 6A,C,E,G, the RMSDs of GABBR2–saikosaponin C, GABBR2–baicalin, NFKBIA–glycyrrhizic acid, and PTGS2–lobetyolin stabilized at approximately 0.7 nm, 1.3 nm, 0.35 nm, and 0.45 nm, respectively. In Figure 6B,D,F,H, the RMSFs in both of the four complexes exhibited minimal fluctuations, all below 1.0, demonstrating limited flexibility within their protein–ligand interactions. Lower radius of gyration (Rg) values indicates tighter protein binding [58]. As shown in Figure S1, the Rg values in both complexes were initially high but stabilized at lower levels after 20 or 30 ns. Solvent accessible surface area (SASA) values evaluate protein stability and folding during the simulation [59]. The SASA changes of the complexes over time are presented in Figure S2. Hbonds significantly influence the stability of the complex because they contribute a major interaction to the complex formation [60]. It was found that the Hbond numbers formed for the GABBR2–saikosaponin C, GABBR2–baicalin, NFKBIA–glycyrrhizic acid, and PTGS2–lobetyolin complexes fluctuated stably between 6 and 8, 4 and 5, 5 and 6, and 3 and 4, respectively (Figure S3). Low RMSD, RMSF, Rg, and SASA values as well as high intermolecular interactions indicated that GABBR2–saikosaponin C, GABBR2–baicalin, NFKBIA–glycyrrhizic acid, and PTGS2–lobetyolin complexes were quite stable, highlighting their potential as promising candidates for fever treatment.

### 2.5. Molecular Mechanics of the Poisson–Boltzmann Surface Area (MMPBSA) Analysis from the MD Simulation Trajectory

Based on the prior MD simulation trajectories, the average binding energies for the GABBR2–saikosaponin C, GABBR2–baicalin, NFKBIA–glycyrrhizic acid, and PTGS2–lobetyolin complexes were computed using the MMPBSA method. The protein–ligand energy components were calculated, including Van Der Waals interaction energy (ΔVDWAALS), electrostatic energy (ΔEEL), polar solvation energy (ΔEGB), and SASA surface energy (ΔESURF) (Table 2). The total binding free energy for the GABBR2–saikosaponin C, GABBR2–baicalin, NFKBIA–glycyrrhizic acid, and PTGS2–lobetyolin complexes were −70.90 ± 4.46 kcal/mol, −55.25 ± 4.40 kcal/mol, −40.89 ± 4.64 kcal/mol and −27.22 ± 4.47 kcal/mol, respectively. Overall, these results were almost identical to those of molecular docking. 

**Figure 7:**
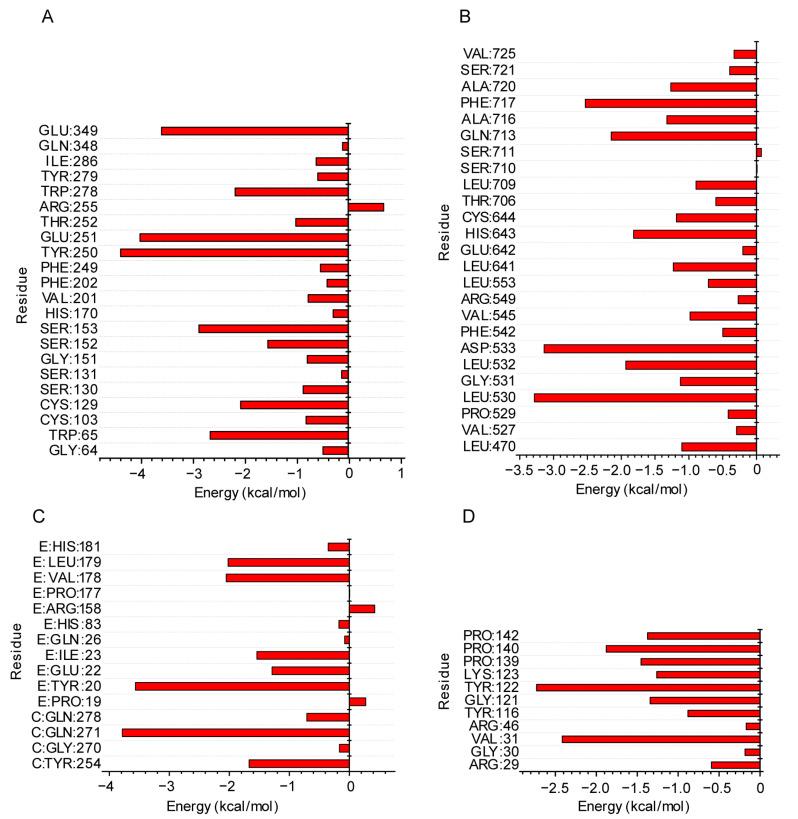
Per-residue energy decomposition for indicated complexes: GABBR2–saikosaponin C (**A**), GABBR2–baicalin (**B**), NFKBIA–glycyrrhizic acid (**C**), and PTGS2–lobetyolin (**D**).

Additionally, key amino acid residues that mainly facilitated protein–ligand binding by decomposing the per-residue energy were assessed. The key residues for ligand–protein interaction in GABBR2–saikosaponin C were TYR65, CYS129, SER152, SER153, TYR250, GLU251, THR252, TRP278, and GLU349 (Figure 7A). The key residues in GABBR2–baicalin were LEU470, LEU530, GLY531, LEU532, ASP533, LEU641, HIS643, CYS644, GLN713, ALA716, PHE717, and ALA720 (Figure 7B). The key residues in NFKBIA–glycyrrhizic acid were TYR254, GLN271, TYR20, GLU22, ILE23, VAL178, and LEU179 (Figure 7C). Furthermore, the key residues in PTGS2–lobetyolin were VAL31, GLY121, TYR122, LYS123, PRO139, PRO140, and PRO142 (Figure 7D). These findings may contribute to the potential for the active compounds to maintain a long-term, stable interaction with the targeted receptors.

### 2.6. XCHG Alleviates Yeast-Induced Fever in Mice

**Figure 8:**
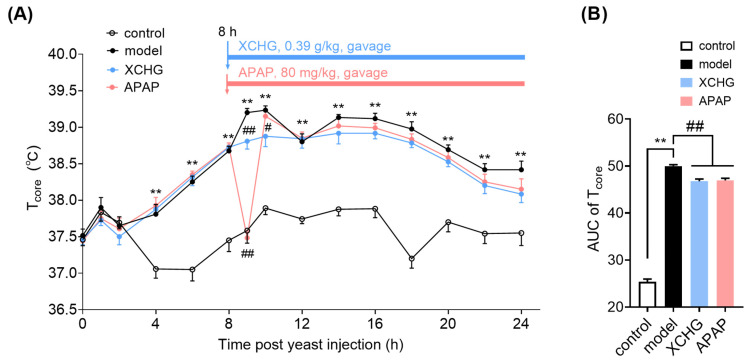
XCHG relieves yeast-induced fever in mice. (**A**) XCHG gavage inhibits the rapid and excessive increase in core body temperature (*T*_core_). (**B**) XCHG gavage significantly decreases the AUC of *T*_core_. Means with different symbols differ significantly: ** *p* < 0.01 vs. control group. ^#^ *p* < 0.05, ^##^ *p* < 0.01 vs. model group. *n* = 12.

The core body temperature (*T*_core_) in the model group was significantly increased from 6 to 24 h compared to the control group (*p* < 0.01) (Figure 8A). Furthermore, the *T*_core_ increased by more than 0.8 °C compared to the initial *T*_core_, confirming the successful construction of the fever model. APAP demonstrated a rapid onset of antipyretic action, restoring feverish mice to a normal *T*_core_ after 1 h of drug administration (Figure 8A), but they reverted to a feverish state within the next hour. Researchers have reported that the mean half-life (T_1/2_) for APAP-exposed mice was 0.46 h [61], and giving APAP in feverish mice caused a ∼2 °C *T*_core_ fall 45 min later [3]. These studies suggest that the results of the present study were consistent with the metabolism characteristics and the antipyretic effect of APAP.

After the administration of XCHG, the rate of *T*_core_ increase in the XCHG group was milder compared to the model group. The *T*_core_ in the XCHG group was lower than that in the model group and the APAP group at 10 h when all drug treatment groups reached their peak temperatures post-dosing. These findings indicate that XCHG can prevent the rapid and excessive increase in *T*_core_ in febrile mice. The area under the curve (AUC) of *T*_core_ over time was used to assess overall changes in *T*_core_ (Figure 8B). The results indicated that yeast significantly increased the AUC whereas XCHG reduced it.

### 2.7. XCHG Reduces IL-1β and IL-8 Expression in Serum

**Figure 9:**
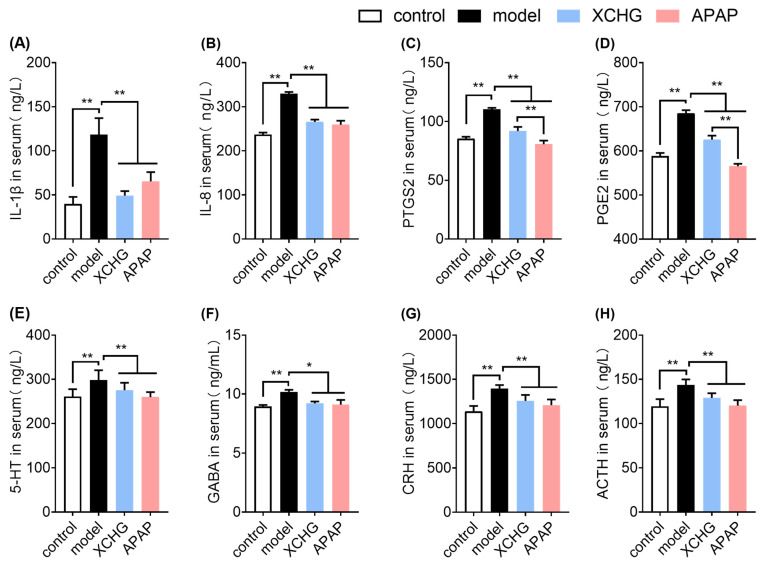
Results of the effect of XCHG on yeast-induced fever in mice. Enzyme-Linked Immunosorbent Assay (ELISA) was used to detect mouse serum levels of IL-β (**A**), IL-8 (**B**), PTGS2 (**C**), PGE2 (**D**), 5-HT (**E**), GABA (**F**), CRH (**G**), and ACTH (**H**). Data are expressed as mean ± SEM (*n* = 8). * *p* < 0.05, ** *p* < 0.01.

NFKBIA, a crucial regulatory gene in the NF-κB signaling pathway, plays a pivotal role in negatively regulating the secretion of downstream regulators IL-1β and IL-8 [62]. To verify the role of NFKBIA in the antipyretic activity of XCHG, we examined the levels of IL-1β and IL-8 in serum. The interleukin family is a key mediator of systemic inflammation, which is often accompanied by fever. In particular, IL-1β and IL-8, which are important mediators of fever and inflammation, are present in whole blood and measurable (in pg/mL) in various systemic inflammatory conditions. Hence, serum concentrations of IL-1β and IL-8 have been suggested to provide insights into certain features of fever-related illnesses [63,64,65]. The levels of IL-1β and IL-8 were notably increased in the model group compared to the control group (Figure 9A,B). Compared to the model group, the XCHG and APAP group exhibited significantly low levels of IL-β and IL-8.

### 2.8. XCHG Inhibits PTGS2 and PGE2 Levels in Serum

Prostaglandins (PGs) are lipid mediators involved in various physiological processes, including the regulation of blood vessel constriction or dilation, inflammation, pain, and fever [66]. PGs are synthesized by nearly all cell types through the activation of PTGS/COX. Inducible PTGS2/COX2 is particularly significant in conditions related to inflammatory signaling. PTGS2/COX2 expression and activation primarily yield PGE2 [67], which significantly increases during tissue damage and inflammation [68]. Additionally, treatment with XCHG resulted in a reduction in PTGS2 and PGE2 levels in the serum (Figure 9C,D). Moreover, the levels of PTGS2 and PGE2 were lower in the APAP group compared to the XCHG group (*p* < 0.05). These findings suggest that XCHG treatment may effectively alleviate the levels of these inflammatory mediators, which is similar to the effect observed with APAP.

### 2.9. XCHG Lowers 5-HT and GABA Levels in Serum

Many neurotransmitters play essential roles in the central regulation of *T*_core_ across various physiological states, including normothermia, heat and cold stress, fever, and exercise. 5-HT, a key mediator in the neuronal system, impacts the *T*_core_ in warm-blooded animals and is abundantly found in the brain, especially in the hypothalamus [69]. Studies have demonstrated that intravenous administration of 5-HT induces an increase in rabbit *T*_core_ [70]. GABA, a common ingredient in sports supplements and other health products, regulates *T*_core_ in the preoptic area and anterior hypothalamus (PO/AH) [71]. Moreover, GABA plays crucial physiological roles in non-neuronal peripheral tissues and organs, including inflammatory modulation [72], immune regulation [73,74], antioxidant effect [75], and antimicrobial effect [76]. In the current study, compared to the model group, APAP and XCHG treatments lowered the levels of 5-HT and GABA in serum (Figure 9E,F).

### 2.10. XCHG Decreases CRH and ACTH Levels in Serum

The febrile response in the brain involves at least two other mediators: CRH and ACTH. The hypothalamic–pituitary–adrenal (HPA) axis can be activated by inflammatory cytokines, such as TNF-α and IL-1β [77]. Notably, the concentration of CRH and ACTH was significantly elevated in the model group compared to the control group (Figure 9G,H). However, APAP and XCHG treatments led to a reduction in CRH and ACTH levels in serum. The above results demonstrated that XCHG may exert a regulatory effect on the HPA axis to alleviate fever.

### 2.11. XCHG Regulates the Hypothalamic Metabolites and Metabolic Pathways

**Figure 10:**
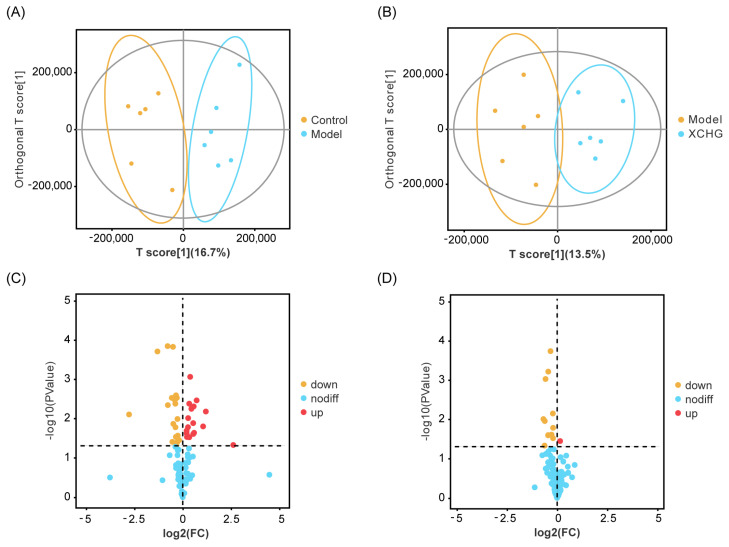

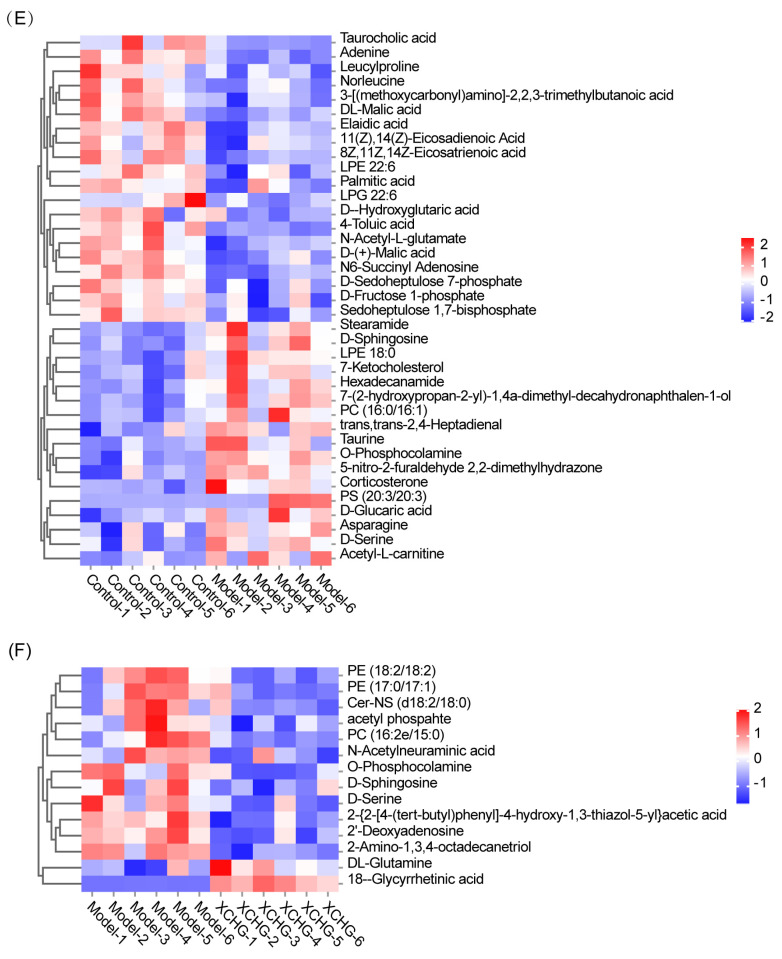
Findings from the analysis of hypothalamic metabolomics. The orthogonal partial least squares discriminant analysis (OPLS-DA) score plot is shown for the control group vs. the model group (**A**) and for the model group vs. the XCHG group (**B**). Volcano plots for the control group vs. the model group (**C**) and for the model group vs. the XCHG group (**D**) are also depicted, where each point symbolizes a metabolite. The size of the point indicates the variable importance in the projection (VIP) value of the metabolite in the OPLS-DA model. The heatmap displays the hierarchical clustering analysis of the differential metabolites for the control group vs. the model group (**E**) and for the model group vs. the XCHG group (**F**). Each column signifies a hypothalamus sample, and each row denotes a differential metabolite. The color gradient, from blue (low levels) to red (high levels), represents the relative level of the differential metabolite. *n* = 6.

The orthogonal partial least squares discriminant analysis (OPLS-DA) revealed notable disparities in metabolic profiles between the model and control groups, as well as the XCHG and model groups (Figure 10A,B). To ensure the robustness of the model, a permutation test was conducted to verify its validity. Metabolites with significant differences in abundance are evident from the volcano plots (Figure 10C,D). Using the OPLS-DA model with variable importance (VIP) > 1.0 and *p* < 0.05 (two-tailed *t*-test), 37 differential metabolites were identified in the model group compared to the control group, whereas 14 differential metabolites were noted in the model group compared to the XCHG group. The generated heatmaps depict the relationships between these differential metabolites in different samples (Figure 10E,F). 

**Figure 11:**
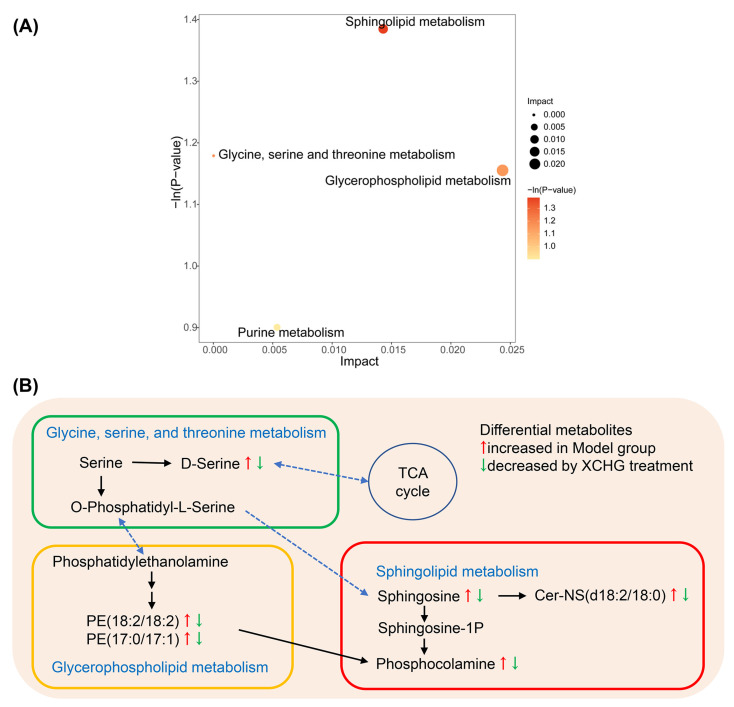
Metabolic network analysis after XCHG treatment. (**A**) Metabolic pathway analysis results based on the 14 regulated hypothalamus metabolites after administration of XCHG. (**B**) Summaries of metabolic pathways.

We employed relative betweenness centrality and the hypergeometric test to identify pathways with important positional information for the 14 differential metabolites between the model and XCHG groups. Notably, XCHG treatment was associated with four metabolic processes (Figure 11A), including sphingolipid metabolism; glycine, serine, and threonine metabolism; glycerophospholipid metabolism; and purine metabolism. Figure 11B summarizes the key differential metabolites, with a particular focus on the role of sphingolipid metabolism in XCHG’s alleviation of yeast-induced fever.

### 2.12. Spearman’s Correlation Analysis of the Detected Indicators

**Figure 12:**
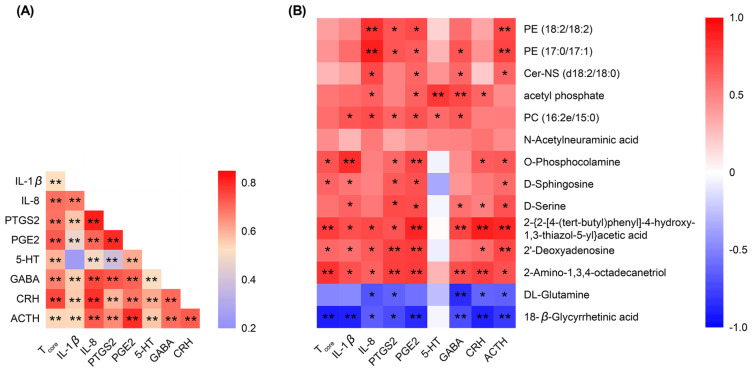
Nonparametric Spearman’s correlation for (**A**) core body temperature (*T*_core_) and serum biochemical indicators and (**B**) *T*_core_, serum biochemical indicators, and the 14 differential metabolites. * *p* < 0.05, ** *p* < 0.01.

To better explore the relationship between *T*_core_ and various biochemical indicators in mice, we performed a Spearman’s correlation analysis. Notably, *T*_core_ was positively correlated with all of the biochemical indicators (Figure 12A). Consequently, the antipyretic effect of XCHG strongly correlated with a decrease in IL-1β, IL-8, PTGS2, PGE2, 5-HT, GABA, CRH, and ACTH levels.

Furthermore, we conducted Spearman’s correlation analysis of pharmacological indicators and differential metabolites in the hypothalamus and calculated the correlation coefficients (Figure 12B). The majority of the differential metabolites correlated with the pharmacological indicators. Mouse *T*_core_, IL-1β, PTGS2, PGE2, and ACTH were significantly positively correlated with D-sphingosine. Cer-NS was positively correlated with IL-8, PGE2, GABA, and ACTH. Pharmacological indicators, except 5-HT, showed a negative correlation with 18-β-glycyrrhetinic acid.

These results indicate a clear link between *T*_core_, serum biochemical indices, and metabolites, aligning with prior research. For example, ellagic acid relieved pyrexia by inhibiting yeast-induced overproduction of IL-1β, PGE2, and metabolic biomarkers PE (16:0/20:4), PC (18:2/15:0), and sphingolipid [78]. Astragali Radix increases *T*_core_ by elevating the cyclic adenosine monophosphate (cAMP) and PGE2 levels, as well as thermo-related metabolites, such as lipids, choline, and leucine [79]. XCHG acts holistically on the blood and hypothalamus to relieve yeast-induced fever, offering strong evidence for the scientific multi-session integrated regulation nature of the property theory in Chinese medicine.

## 3. Discussion

The chemical composition of TCM is complex. Therefore, analyzing the chemical composition of TCM can better clarify the basis of the medicinal effects. In this study, the chemical composition of XCHG was analyzed using UFLC-Q-TOF-MS/MS. The main components of XCHG were flavonoids and triterpenoid saponins. Flavonoids have been demonstrated to have a considerable influence on the immune system and inflammatory reactions [80,81]. Various flavonoids in XCHG, including quercetin, baicalin, apigenin, and glycyrrhizin, have been demonstrated to possess anti-inflammatory activity. Quercetin in particular stands out for its anti-inflammatory and immunoregulatory activities. It decreases the levels of IL-1β, TNF-α, and IL-8. Additionally, quercetin inhibits the activity of COX both in vitro and in vivo [82,83]. Triterpenoid saponins detected in XCHG, including saikosaponin B1, saikosaponin B2, saikosaponin C, saikosaponin A, and glycyrrhizic acid, have also been reported to possess antipyretic and anti-inflammatory activities [84]. Saikosaponin B2 has been demonstrated to suppress the release of PGE2, TNF-α, and IL-1β in inflammatory responses [85]. Glycyrrhizic acid has been reported to improve inflammation via the activated glucocorticoid receptor and the Phosphonosinol-3 Kinase/Protein Kinase B/glycogen synthase kinase 3β (PI3K/AKT/GSK3β) signaling pathway [86]. Additionally, glycyrrhizic acid exerts a neuroprotective effect in the postischemic brain by inhibiting HMGB1 phosphorylation and secretion [87]. Curcumin exerts its antipyresis by inhibiting the glutamate–hydroxyl radical–PGE2 pathways in the hypothalamus and circulating TNF-α, IL-1β, and IL-6 levels [88]. These findings underscore the diverse constituents present in XCHG, suggesting a holistic approach to addressing inflammation and fever through multiple bioactive compounds acting synergistically.

Chinese medicines are distinguished by multi-target and holistic actions. Molecular docking and MD techniques serve as crucial tools for predicting the binding types and interaction configurations of biomacromolecular complexes, offering valuable references and theoretical backing for further experiments [89]. Molecular docking, a common method in network pharmacology, involves identifying the primary and most effective compounds from molecular databases through a scoring system, thus markedly improving screening efficiency over conventional approaches [90]. MD techniques involve a computational approach for assessing binding stability by simulating the dynamics of protein–ligand complexes arising from molecular docking [91]. In this study, we employed network pharmacology, molecular docking, and MD to analyze XCHG’s potential active compounds and fever-related targets. Notably, XCHG’s antipyretic activity was associated with four modules: inflammation/immune response, neuromodulation, metabolism, and vasodilation. Additionally, different active ingredients exhibited different interactions with the key targets, implying the possibility that the active ingredients in XCHG act synergistically to alleviate fever. Saikosaponin C, saikosaponin A, and glycyrrhizic acid demonstrated a robust affinity for GABBR2. GABA, the most common inhibitory neurotransmitter in the central nervous system, could specifically bind to two GABA receptors (GABA-A and GABA-B receptors) [92]. Moreover, baicalin exhibited a strong affinity for GABA-A receptors, along with anti-inflammatory and anxiolytic effects [48]. Saikosaponin C, saikosaponin A, glycyrrhizic acid, and liquiritin demonstrated robust binding energy to NFKBIA. Lobetyolin, the bioactive constituent in codonopsis radix extracts, had the lowest binding energy among all of the active compounds that bind to PTGS2. Lobetyolin exerts anti-inflammatory, antioxidant, and antitumor activities [93]. Considering the role of each medicinal plant part in XCHG, along with compound content and docking scores, we selected specific protein–ligand complexes, including GABBR2–saikosaponin C, GABBR2–baicalin, NFKBIA–glycyrrhizic acid, and PTGS2–lobetyolin, for MD simulations to assess their stability. RMSD, RMSF, Rg, SASA, and Hbond numbers indicated that the selected complexes had high stability, compactness, and flexibility. Additionally, high inter-molecular interactions were observed during the 100 ns MD simulation process. The totals of average binding free energies of the indicated complexes were negative, indicating that all proposed molecules exhibited stable binding affinity towards the corresponding targets. Notably, saikosaponin C and baicalin were bound to the N-terminal and C-terminal in GABBR2, suggesting that the two ligands may act synergistically on this protein to exert antipyretic effects. The predictable robust interplay between these active ingredients and their intended targets forms the foundation for studying the biological activity of these molecules, suggesting their potential as promising candidates for fever treatment. However, it should be noted that the accuracy of the calculation of binding free energy involves many uncertain factors, such as dielectric constant, ligand formal charge, and the number of protein folds [94]; further experiments are needed to further verify the ligand–protein interactions.

The mouse fever model, induced by subcutaneous injection of dry yeast, is commonly used to study the mechanisms of fever and assess the efficacy of antipyretic drugs [95]. Dry yeast can activate the immune system, leading to the release of cytokines, such as IL-1β and TNF-α [96]. These immune-mediated responses result in inflammation and an increase in *T*_core_, aiding the body’s defense against pathogens [97]. Although the precise mechanisms of yeast-induced fever remain unclear, it may involve the regulation of adipose tissue and energy metabolism [98]. We observed that XCHG treatment effectively reduced the levels of inflammatory factors, pyrogenic mediators, neurotransmitters, and endocrine markers in mice. The ELISA demonstrated that IL-1β and IL-8 levels in the XCHG group were significantly lower than those in the model group, suggesting that XCHG exerts its antipyretic effect by inhibiting the expression of IL-1β and IL-8, two key mediators in the febrile response.

PGE2, a key mediator in fever induction, is synthesized from arachidonic acid generated from cell membrane phospholipids [99]. Several studies have demonstrated that many endocrine tissues respond to PGE2, with systemic administration leading to elevated circulating levels of ACTH [100]. We observed that XCHG treatment significantly reduced the levels of PTGS2 and PGE2 in febrile mice. XCHG also reduced CRH and ACTH levels. These findings indicate that XCHG alleviates fever by inhibiting PTGS2 and PGE2 synthesis and progressively modulating CRH and ACTH to reset the *T*_core_ of febrile mice to a lower set point.

The sphingolipid signaling pathway, one of the most crucial secondary signaling systems within cells [101], stimulates specific sphingosine kinases to generate sphingosine-1-phosphate (S1P), a key molecule in the sphingolipid signaling pathway [102]. Inflammatory stimuli can accelerate the production of sphingolipids, resulting in sphingolipase-induced production of ceramide (Cer) and sphingosine. Additionally, ACTH stimulation can catalyze the conversion of sphingolipids into sphingosine [103]. Sphingosine, an essential component of cell membranes, is a biologically active signaling molecule. Various cytokines, including TNF-α and IL-1β, can activate sphingosine kinase (SphKs), thus catalyzing the phosphorylation of sphingosine to produce S1P, which plays a role in regulating immune responses and inflammatory processes [104]. XCHG may exert its effects by inhibiting IL-1β, consequently suppressing SphKs and S1P formation. This process helps in regulating immune responses and inflammation, contributing to reducing fever. 

To conclude, XCHG’s efficacy in treating fever could be linked to functional modules, including inflammation/immune, neuromodulation, and metabolism. The effect may primarily involve the interaction of the key active compounds (saikosaponin C, baicalin, glycyrrhizic acid, and lobetyolin) with core targets (GABBR2, NFKBIA, and PTGS2). Animal studies indicated that XCHG could alleviate yeast-induced fever and inhibit the rapid and excessive increase in *T*_core_. The amelioration of symptoms was primarily associated with the regulation of inflammatory factors (IL-1β and IL-8), pyrogenic mediators (PTGS2 and PGE2), neurotransmitters (5-HT and GABA), and endocrine markers (CRH and ACTH). Meanwhile, XCHG influenced 14 endogenous metabolites in the hypothalamus, including sphingosine, Cer-NS, phosphatidylethanolamine (PE), phosphatidylcholine (PC), O-phosphocolamine, and 18-β-glycyrrhetinic acid. These findings provide valuable insights into the potential antipyretic mechanisms of XCHG. The drawback of this study is that we did not determine how the active ingredients (saikosaponin C, baicalin, glycyrrhizic acid, and lobetyolin) of XCHG interact with the core target proteins (GABBR2, NFKBIA, and PTGS2). Further research is required to illustrate the exact molecular mechanisms and pathways underlying XCHG’s antipyretic effects.

## 4. Materials and Methods

### 4.1. Materials and Reagents

Seven medicinal plant components, including CH (Batch No. 12006B014), HQ (Batch No. 12010B019), DS (Batch No. 12010B018), GC (Batch No. 12004B003), DZ (Batch No. 12010B024), JBX (Batch No. 12010B010), and SJ (Batch No. 12011B014), were provided by Guangzhou Baiyunshan Guanghua Pharmaceutical Co., Ltd. (Guangzhou, China). The preparation process for XCHG extracts was as follows: (1) in accordance with the compound’s proportions, as described in the *Chinese Pharmacopoeia* [17], the recommended amount of medicinal materials in XCHG was weighed; (2) five of the medicinal plants (CH, HQ, DS, GC, and DZ) were decocted twice with water, and the liquids were mixed and filtered; (3) the resulting filtrate was concentrated; (4) the other two medicinal plants (JBX and SJ) were macerated with 70% ethanol for 24 h and percolated, and the ethanol was recovered; (5) the percolate was combined with the concentrate obtained earlier; (6) the mixture was then concentrated using a rotary vacuum evaporator (Eyela N-1100, Shanghai, China), followed by lyophilization using a freeze dryer (Christ alpha 1-4 LD plus, Osterode, Germany); and (7) the final extracts were stored at −80 °C until further utilization.

Chemical reference standards were purchased from several companies, including the National Institutes for Food and Drug Control (Beijing, China), Zhongshan CN-Biotechnology Co., Ltd. (Zhongshan, China), and Shanghai yuanye Bio-Technology Co., Ltd. (Shanghai, China) (Table S1).

### 4.2. Chemical Composition of XCHG

#### 4.2.1. Sample Pretreatment

To analyze the distribution of chemical constituents within the herbs comprising XCHG, we simulated the XCHG preparation process by preparing extracts of each of the seven herbs. In brief, five herbs (CH, HQ, DS, GC, and DZ) were separately extracted with water twice and concentrated to obtain five single-herb extracts. The remaining two herbs (JBX and SJ) separately underwent a 24 h immersion in 70% ethanol, followed by percolation to obtain JBX and SJ extracts after the recovery of ethanol. The final extracts were stored at −80 °C. The stock solutions of 34 reference standards were dissolved in methanol to a final concentration of 10 μg/mL and stored at 4 °C until analysis.

The XCHG extracts (0.02 g) and seven single-herb extracts (1.5 g) were subjected to ultrasonic extraction using a 70% methanol–water solution (*v*/*v*, 20 mL) for 20 min and then adjusted to a constant volume of 25 mL. Additionally, [2′,5′,6′,6,8-D5] quercetin (used as an internal standard) was added to the test solutions to achieve a final concentration of 500 ng/mL.

#### 4.2.2. UFLC-Q-TOF-MS/MS Analysis

A UPLC (Shimadzu Corp. Kyoto, Japan)-hybrid triple quadruple time-of-flight mass spectrometer (Triple TOF™ 5600+, AB Sciex, Forster City, CA, USA) with an electrospray ionization source (ESI) was used in the current study. Chromatographic separation was carried out on a Kinetex^®^ C18 column (3.0 × 150 mm, 2.6 μm, Phenomenex) at a flow rate of 0.3 mL/min. The column temperature was maintained at 40 °C. The injection volume was 3 μL. The mobile phase consisted of acetonitrile (A) and 0.1% formic acid (B). A multi-step linear gradient elution was applied: 5–50% A at 0–30 min, 50–95% A at 31–40 min, and 5% A at 41–45 min.

ESI was employed for MS/MS identification. The ion spray voltage floating was set at 4500 V in negative ion mode and 5500 V in positive ion mode. The mass-to-charge ratio (*m*/*z*) ranged from 50 to 1500 Da. The ion source temperature was maintained at 550 °C. In both modes, gas source 1 (GS1) and gas source 2 (GS2) were set to 55 psi, and curtain gas pressure was 35 psi. The declustering potential (DP) was 80 V, while collision energy and its spread were 30 eV and 15 eV, respectively. Nitrogen served as the nebulizing and auxiliary gas. Data acquisition was performed using the Analyst^®^ TF 1.6 software (AB Sciex, Foster City, CA, USA) in the information-dependent acquisition mode.

#### 4.2.3. Quantification Analysis

For quantification analysis, 9 mg of the XCHG extract was dissolved in 9 mL of 70% ethanol in a 10 mL volumetric flask and sonicated for 20 min. The solution was then made up to volume with 70% ethanol. Subsequently, the mixture was further diluted to an appropriate concentration and filtered through a 0.22 µm filter for UFLC-Q-TOF-MS/MS analysis. Stock solutions of baicalin, baicalein, wogonin, wogonoside, quercetin, saikosaponin B1, saikosaponin B2, glycyrrhizic acid, and liquiritin were prepared in methanol and serially diluted using 70% methanol. The external standard curve method was employed to quantify the compounds. Chromatographic separation and mass spectrum parameters were the same as previously described in Section 4.2.2.

### 4.3. Network Analysis

#### 4.3.1. Target Prediction and Module Network Construction

The active compounds and their corresponding targets were screened using Traditional Chinese Medicine Systems Pharmacology (TCMSP) (http://tcmspw.com/tcmsp.php, accessed on 15 June 2023), SwissADME (http://www.swissadme.ch/, accessed on 15 June 2023), and SwissTargetPrediction Database (http://www.swisstargetprediction.ch/, accessed on 15 June 2023), following previously reported methods [105]. The targets of the ingredients were standardized using the UniProt database (https://www.uniprot.org, accessed on 18 June 2023). Disease targets were searched using the keywords “fever”, “cold fever”, and “flu fever” through the GeneCard (https://www.genecards.org/, accessed on 18 June 2023) and Online Mendelian Inheritance in Man (OMIM) databases (https://omim.org/, accessed on 15 June 2023). The intersection of XCHG targets with fever targets was determined using Venny (Version 2.1), with the resulting targets considered potential antipyretic targets of XCHG.

The herb–compound–biological functional module network construction followed a previously described method [106]. Gene Ontology (GO) enrichment and *Kyoto Encyclopedia of Genes and Genomes* (KEGG) pathway analysis of XCHG–fever intersecting targets were conducted using the DAVID database (https://david.ncifcrf.gov/, accessed on 23 June 2023). Significantly enriched KEGG pathways and GO biological processes were categorized into inflammation/immune, neuromodulation, metabolism, and vasodilatory processes. Compounds considered to be main active compounds were (1) those that are for determination of this herb documented in the *Chinese Pharmacopoeia* (2020 edition); (2) those frequently reported in the literature or that are pharmacologically active; and (3) those that can significantly enrich at least one key GO biological process/KEGG signaling pathway related to XCHG. Genes were selected if (1) the gene expression can characterize the changes in biological functional modules; (2) they are associated with the pathogenesis of fever and XCHG’s effect; and (3) they are associated with significantly enriched biological processes or signaling pathways (*p* < 0.01). The STRING database was used to examine protein–protein interactions (PPI) for the selected genes, and Cytoscape 3.7.2 was employed to visualize the network. If one gene was linked to another in a PPI network, then it was considered to be connected. A herb was connected to a biological functional module if it had been reported to affect this biological functional module. Biological functional modules were connected if they shared genes in associated KEGG signaling pathways/GO biological processes. Active compounds of the respective herbs were also connected in the network.

#### 4.3.2. Molecular Docking

The top-ranked PPI targets were selected from each of the four biological functional modules within the herb–compound–biological functional module network. The main active compounds of XCHG responsible for the antipyretic effect were selected as ligands for molecular docking. The 3D structures of these compounds were downloaded from PubChem (https://pubchem.ncbi.nlm.nih.gov/ accessed on 15 September 2023). Protein receptor files were obtained from the UniProt database (http://www.rcsb.org/, accessed on 15 September 2023). These receptor proteins underwent various pre-processing steps using AutoDock Tools. These steps included removing crystallographic water, eliminating non-standard peptide segments, merging non-polar hydrogens, combining lone pairs of electrons, and retaining only the protein structure while preserving its protonation state.

Molecular docking and result calculations were carried out using AutoDock Vina with the QuickVina-W algorithm. The resulting docking complexes were transformed into PDB format files using Open Babel 3.1.1 and subsequently used to generate ligand–receptor binding illustrations with PyMOL 2.6.0a0.

#### 4.3.3. MD Simulation

To confirm the stability of the protein–compound complexes identified in the docking outcomes, those with the top binding scores were selected for MD simulations using Gromacs 2022.4 for 100 ns. Protein topology was created using the Amber14sb force field and TIP3P water model, whereas ligands were parameterized using the ACPYPE tool [107]. Subsequently, energy minimization was accomplished using the steepest descent for 10,000 steps. Equilibration was then conducted using NVT and NPT at 300 K [108]. Trajectories were recorded every 10 ps and analyzed using Origin 85 programs. Various parameters were calculated to assess the stability of the complexes, including root mean square deviation (RMSD), root mean square fluctuation (RMSF), radius of gyration (Rg), solvent accessible surface area (SASA), and hydrogen bond (Hbond) number. These parameters collectively characterize the protein–ligand’s stability, flexibility, compactness, hydrophobicity, and binding strength.

#### 4.3.4. Molecular Mechanics Poisson–Boltzmann Surface Area (MMPBSA) Calculation

Thermodynamic parameters of the protein–ligand complexes were analyzed using the MMPBSA method in gmx_MMPBSA v.1.6.2 software [109,110]. Based on the kinetic simulation trajectories, snapshots at 100 ps intervals ranging from 80 ns to 100 ns MD trajectories were obtained to calculate their binding free energies with the standard deviation (SD) using the following equation:

Δ*G_bind_* = (*G_COM_*) − (*G_REC_*) − (*G_LIG_*)

*G_COM_*, *G_REC_*_,_ and *G_LIG_* denote the total free energy of the protein–ligand complex, the receptor protein, and the ligand, respectively. Both polar and nonpolar solvation energies were also calculated [110].

### 4.4. Experimental Verification

#### 4.4.1. Animal Experimental Design

Eight-week-old male C57 black mice were acquired from the Laboratory Animal Center of Sun Yat-sen University. All procedures involving animal housing and experimentation adhered to the local animal welfare and use guidelines. The animal use protocols were reviewed and approved by the Institutional Animal Care and Use Committee (IACUC), Sun Yat-Sen University (Approval No. SYSU-IACUC-2022-000022). The mice were randomly divided into four groups: control, model, XCHG, and APAP (positive control).

Two days before the experiment, the core body temperature (*T*_core_) of the mice was measured twice daily, in the morning and in the evening, by inserting a thermometer probe (FT3400, Nanjing Calvin Biotechnology Co., Ltd., Nanjing, China) into the mouse’s rectum. The average *T*_core_ within the two days was considered the initial *T*_core_. The pyrexia mouse model was established following the protocol described by Liu et al. (2017) [111]. Briefly, fever was induced by subcutaneously injecting 20% yeast suspension into the dorsum at a volume of 10 mL/kg body weight. The control group received an equivalent volume of saline. At 8 h post yeast injection, the XCHG group received XCHG extracts through gavage administration at a dose of 0.39 g/kg, whereas the APAP group received an dose of APAP (80 mg/kg) by gavage. The *T*_core_ was recorded at 14 time points (1, 2, 4, 6, 8, 9, 10, 12, 14, 16, 18, 20, 22, and 24 h) after the yeast injection. Mice exhibiting a temperature increase exceeding 0.8 °C were classified as having a fever [112,113].

At 9 h post yeast injection, blood samples were collected from the orbit and centrifuged at 3000 rpm/min for 10 min to obtain the serum. Immediately after blood sample collection, the mice were sacrificed through cervical dislocation. The hypothalamus was swiftly isolated, frozen in liquid nitrogen, and stored at −80 °C.

#### 4.4.2. ELISA Analysis of Serum Biochemical Indicators

Enzyme-Linked Immunosorbent Assay (ELISA) kits were purchased from Meimian Biotechnology, Jiangsu, China. The serum concentrations of IL-1β, IL-8, PGE2, PTGS2, 5-HT, GABA, CRH, and ACTH were analyzed following the manufacturer’s instructions for corresponding ELISA kits.

#### 4.4.3. Untargeted Metabolomics Analysis of the Hypothalamus

Hypothalamic tissues (100 mg) were ground with liquid nitrogen, and the homogenate was vortexed with 500 μL of prechilled 80% methanol. Subsequently, the mixture was incubated on ice for 5 min, followed by centrifugation at 15,000 g at 4 °C for 20 min. The supernatant was diluted with LC-MS-grade water to achieve a final concentration containing 53% methanol. After another centrifugation step at 15,000 g/min at 4 °C for 20 min, the supernatant was collected for UHPLC-MS/MS analysis, and 50 μL of each sample was taken and mixed as a quality control sample.

The UHPLC-MS/MS analysis was conducted using the Vanquish UHPLC system (Thermo Fisher, Bremen, Germany) coupled with an Orbitrap Q Exactive^TM^ HF-X mass spectrometer (Thermo Fisher, Bremen, Germany). Chromatographic separation was carried out using a Hypesil Gold column (100 × 2.1 mm, 1.9 μm) at a column temperature of 40 °C. The mobile phase consisted of 0.1% formic acid (A) and methanol (B). The gradient elution program was as follows: 2% B (0–1.5 min), 2–85% B (1.5–3 min), 85–100% B (3–9 min), 100–2% B (9–9.1 min), and 2% B (9.1–12 min). The flow rate was 0.2 mL/min. Mass spectrometry was carried out in positive/negative polarity mode with a spray voltage of 3.2 kV, capillary temperature of 320 °C, sheath gas flow rate of 40 arb, and aux gas flow rate of 10 arb.

The raw data files were imported into Compound Discoverer 3.1 (CD3.1, Thermo Fisher) for peak alignment, peak picking, and quantitation for each metabolite. The primary parameter settings were as follows: retention time tolerance, 0.2 min; signal intensity tolerance, 30%; signal/noise ratio, 3; actual mass tolerance, 5 ppm; and minimum intensity, 100,000. The peak intensities were normalized to the overall spectral intensity. The normalized data were then used to estimate the chemical formula by considering additive ions, molecular ion peaks, and fragment ions. After that, the peaks were aligned using the mzCloud (https://www.mzcloud.org/, accessed on 8 October 2023), mzVault, and Masslist databases to gain accurate qualitative and relative quantitative results. The data matrix generated was then imported into SIMCA-P (Version 14.1) (Umetrics, Umea, Sweden) for orthogonal partial least square–discriminant analysis (OPLS-DA). Metabolites with a variable importance in projection (VIP) score greater than 1 and a *p*-value less than 0.05 (significance test) between groups were considered differential metabolites. Pathway enrichment analysis was performed using MetaboAnalyst and the KEGG (http://www.genome.jp/kegg/, accessed on 10 October 2023) database.

### 4.5. Statistical Analysis

All statistical analyses were performed using GraphPad Prism 8.0.1 (GraphPad Prism Software, La Jolla, CA, USA). The data are presented as means ± standard error of the mean (SEM) for each group. One-way ANOVA followed by Tukey’s test were performed to evaluate the differences between the groups. *p* < 0.05 was considered significant. The correlation between *T*_core_ and serum biochemical indicators was explored using a nonparametric Spearman’s correlation test. Similarly, the correlation between pharmacological indicators (*T*_core_ and serum biochemical indicators) and 14 differential metabolites was also explored. The resultant correlation matrix was visualized using a heatmap.

## Figures and Tables

**Table 1:** Herbal pharmacognostic potential and chemical composition.

Plant Name	Pharmacognostic Potential	Chemical Composition
*Bupleurum chinense* DC.	Antioxidant, antipyretic, immunoregulation, antitumor, hepatoprotection, anti-inflammatory, antiarrhythmic, antifatigue [21,22]	Flavonoids (quercetin, luteolin, apigenin-8-C-β-D-glucoside), triterpenoid saponins (saikosaponins A, C, and D), phenylpropanoids (ferulic acid), coumarins [23,24]
*Scutellaria baicalensis* Georgi	Antiviral, anti-allergic, antitumor, anti-bacterial, antioxidant, anti-inflammatory, hepatoprotective, and neuroprotective activities [25,26]	Flavonoids (baicalin, baicalein, wogonin, wogonoside), phenylethanoid glycosides (martynoside) [25,27]
*Pinellia ternata* (Thunb.) Makino	Sedative, hypnotic, and anticonvulsant activities [28], antitumor, antitussive, antiasthmatic, anti-gastric ulcer, and antidiarrheal effects [29]	Alkaloids (L-Tyrosine, Guanosine, Adenosine), flavonoids (baicalin, Daidzein), phenylpropanoids (Coniferin), others (gingerol) [29,30]
*Codonopsis pilosula* (Franch.) Nannf.	Neuroprotective, immunomodulatory, antitumor, anti-inflammatory, antioxidant, hepatoprotective, anti-hypoxia, antifatigue, and prebiotic activities [31]	Saccharides (rhamnose, arabinose, oligosaccharides, polysaccharides), alkaloids (codonopyrrolidiums A, B), glycosides (lobetyolin), amino acids, [31,32]
*Zingiber officinale* Roscoe	Immunomodulatory, antitumorigenic, anti-inflammatory, anti-apoptotic, anti-hyperglycemic, anti-lipidemic and anti-emetic actions [33]	Gingerols {[4]-, [6]-, [7]-, [8]-, and [10]-gingerol, 6-gingerol, 6-shogaol, [4]-, [6]-, [8]-, [10]- and [12]-shogaol}, volatile oil (curcumene, terpineol, borneol) [33]
*Glycyrrhiza uralensis* Fisch. ex DC.	Antioxidant, anti-inflammatory, antiviral, antidiabetic, skin-whitening, and cholinergic activities [34]	Phenolic (kaempferol, Gancaonin I, Liquiritigenin, Formononetin), flavonoids (Schaftoside, liquiritin, Isoliquiritin), and triterpenoid saponins (glycyrrhizin, glycyrrhetinic acid) [34]
*Ziziphus jujuba* Mill.	Anticancer, antioxidant, anti-inflammatory, anti-hyperlipidemic, anti-hyperglycemic, immunoregulatory, neuroprotective, sedative, and antiviral functions [35]	Polyphenols (Gallic acid, Caffeic acid, Chlorogenic acid, Rutin), polysaccharides, amino acids, nucleotides, fatty acids, dietary fiber, alkaloids [35]

**Table 2:** Average binding energy calculation of protein–ligand complexes using MMPBSA calculation implemented in gmx_MMPBSA.

Complexes	Average Binding Energy (kcal/mol)				
	ΔVDWAALS	ΔEEL	ΔEGB	ΔESURF	ΔTOTAL
GABBR2_6wiv-saikosaponinc	−56.62 ± 4.36	−102.76 ± 7.25	96.44 ± 4.61	−7.96 ± 0.3	−70.90 ± 4.46
GABBR2_6wiv-baicalin	−55.51 ± 3.58	−55.55 ± 9.36	63.14 ± 5.26	−7.33 ± 0.22	−55.25 ± 4.40
NFKBIA_1ikn-glycyrrhizicacid	−53.99 ± 2.57	−44.82 ± 8.35	64.53 ± 5.85	−6.61 ± 0.25	−40.89 ± 4.64
PTGS2_AF_P35354-lobetyolin	−34.62 ± 3.97	−29.21 ± 11.19	41.89 ± 8.05	−5.27 ± 0.55	−27.22 ± 4.47

Footnote: The energy component of binding free energy comprises Van Der Waals interaction energy (ΔVDWAALS), electrostatic energy (ΔEEL), polar solvation energy (ΔEGB), and SASA surface energy (ΔESURF). ΔTOTAL denotes the total binding free energies.

## Data Availability

Data are contained within the article and the Supplementary Materials.

## Supplementary Materials

The following supporting information can be downloaded at: https://www.mdpi.com/article/10.3390/ph17040475/s1, Table S1: The general information of reference standards; Table S2: Identification of chemical components in XCHG through UFLC-Triple TOF-MSMS; Table S3: Results of calibration curve and content for the determination of 9 compounds; Table S4: The active ingredients of XCHG; Table S5: Enriched KEGG signaling pathway/GO biological process of XCHG fever targets; Figure S1: Radius of gyration (Rg) values of indicated complexes; Figure S2: Solvent accessible surface area (SASA) values of indicated complexes; Figure S3: Hydrogen bond (Hbond) numbers of indicated complexes. References [81,114,115,116,117,118,119,120,121,122,123,124,125,126,127,128,129,130,131,132,133,134,135,136,137,138,139,140,141,142] are cited in the Supplementary Materials.

## Abbreviations

5-HT	5-hydroxytryptamine
ACTH	Adrenocorticotrophin
ADME	Absorption, distribution, metabolism, and excretion
APAP	Acetaminophen
AUC	Area under the curve
BPCs	Basic peak chromatograms
Cer	Ceramide
Cer-NS	Ceramide-N-(tetracosanoyl)-sphingosine
CH	Dried root of *Bupleurum chinense* DC. [Apiaceae]
COVID-19	Coronavirus disease 2019
COX	Cyclooxygenase
CRH	Corticotropin releasing hormone
CXCL8	Interleukin-8
DL	Drug-likeness
DS	Dried root of *Codonopsis pilosula* (Franch.) Nannf. [Campanulaceae]
DZ	Dried ripe fruit of *Ziziphus jujuba* Mill. [Rhamnaceae]
EGFR	Epidermal growth factor receptor
ELISA	Enzyme-Linked Immunosorbent Assay
ESR1	Estrogen receptor 1
GABA	γ-aminobutyric acid
GABBR1	γ-aminobutyric acid B receptor 1
GABBR2	γ-aminobutyric acid B receptor 2
GABRG2	GABA-A receptor
GC	Dried root of *Glycyrrhiza uralensis* Fisch. Ex DC. [Fabaceae]
GO	Gene ontology
Hbonds	Hydrogen bonds
HQ	Dried root of *Scutellaria baicalensis* Georgi [Lamiaceae]
IL	Interleukin
JBX	Ginger-processed *Pinellia ternata* (Thunb.) Makino [Araceae]
KEGG	*Kyoto Encyclopedia of Genes and Genomes*
MD	Molecular dynamics
MMPBSA	Molecular mechanics Poisson–Boltzmann surface area
NFKBIA	NF-κB inhibitor IκBα
NF-κB	Nuclear factor kappa-B
OB	Oral bioavailability
OPLS-DA	Orthogonal partial least squares discriminant analysis
PC	Phosphatidylcholine
PE	Phosphatidylethanolamine
PGE2	Prostaglandin E2
PGs	Prostaglandins
PPI	Protein–protein interactions
PTGS	Prostaglandin endoperoxide synthases
Rg	Radius of gyration
RMSD	Root mean square deviation
RMSF	Root mean square fluctuation
S1P	Sphingosine-1-phosphate
SASA	Solvent accessible surface area
SJ	Fresh root of *Zingiber officinale* Roscoe [Zingiberaceae]
SphKs	Sphingosine kinase
SSs	Saikosaponins
STAT3	Signal transducer and activator of transcription 3
TCM	Traditional Chinese medicine
*T*_core_	Core body temperature
TNF	Tumor necrosis factor
UFLC-Q-TOF-MS/MS	Ultra-fast liquid chromatography/quadrupole-time-of-flight tandem mass spectrometry
UHPLC-MS	Ultra-high-performance liquid chromatography–tandem mass spectrometry
VEGFA	Vascular endothelial growth factor A
