# Supplementary material for: Unraveling the Mechanism of Xiaochaihu Granules in Alleviating Yeast-Induced Fever Based on Network Analysis and Experimental Validation
Source: Pharmaceuticals (Basel). 2024 Apr 8;17(4):475. doi: 10.3390/ph17040475 (PMC11053540; doi:10.3390/ph17040475)
Supplement: Supplementary file 1 [file pharmaceuticals-17-00475-s001.zip › pharmaceuticals-2913698-supplementary.pdf]

Supplementary Material

Unraveling the Mechanism of Xiaochaihu Granules in Alleviating Yeast-Induced Fever Based on Network Analysis and Experimental Validation

Xiuli Chen, Hao Wu, Peibo Li, Wei Peng, Yonggang Wang, Xiaoli Zhang, Ao Zhang, Jinliang Li, Fenzhao Meng, Weiyue Wang and Weiwei Su*

Guangdong Engineering & Technology Research Center for Quality and Efficacy Reevaluation of Post-Market Traditional Chinese Medicine, Guangdong Provincial Key Laboratory of Plant Resources, State Key Laboratory of Biocontrol, School of Life Sciences, Sun Yat-sen University, Guangzhou 510275, China

* Correspondence: lsssww@126.com

Table S1 The general information of reference standards

No.	Compounds	Batch number	Source
1	Baicalin	715-200010	a
2	Citric acid	100396-201302	a
3	Baicalein	111595-201808	a
4	Wogonoside	DST190709-026	b
5	Wogonin	111514-201706	a
6	Scutellarin	110842-200403	a
7	Succinic acid	110896-200001	a
8	Calycosin-7-*O*-β-D-glucoside	111920-201606	a
9	Formononetin	C-018-181216	b
10	Chlorogenic acid	110753-201415	a
11	Neochlorogenic acid	P20A11L121936	c
12	Chrysin	111701-200501	a
13	Rutin	110080-9705	a
14	Liquiritigenin	F1912131	f
15	Isoliquiritigenin	F2001050	f
16	Isoliquiritin	Y15A10H95344	c
17	Schaftoside	Z25O10B101118	c
18	Isoschaftoside	P13D11S134210	c
19	Liquiritin apioside	5569	i
20	Saikosaponin c	M12O11S126866	c
21	Caffeic acid	110885-200102	a

No.	Compounds	Batch number	Source
22	Saikosaponin A	150102	e
23	Saikosaponin B1	P09M10F82675	c
24	Saikosaponin B2	Z04S9L69482	c
25	L-Malic acid	191123-038	b
26	Phenprobamate	B21910	h
27	Proline	BCBZ5040	g
28	Glutamic acid	111576-200201	a
29	Aspartic acid	140691-201602	a
30	Arginine	140685-201305	a
31	Liquiritin	111610-201106	a
32	Glycyrrhizic Acid	P24J10F91300	c
33	Glycyrrhetinic Acid	471-53-4	d
34	Lobetyolin	220321	e

a: National Institutes for Food and Drug Control. b: Zhongshan CN-Biotechnology Co., Ltd. c：Shanghai yuanye Bio-Technology Co., Ltd. d：Beijing Qisong Biotechnology Co., Ltd. e：Zhongshan UNO Biotechnology Development Co., Ltd. f：Shanghai Aladdin Biochemical Technology Co., Ltd. g：Sigma-Aldrich. h：Guangzhou Meilun Biotechnology Co., Ltd. I: Nature standard

Table S2 Identification of chemical components in XCHG by UFLC-Triple TOF-MSMS

No.	formula	t_R, min	$[M+H]^+$ (error, ppm)	$[M-H]^-$ (error, ppm)	Major fragment ions in positive mode (m, z) [b]	Major fragment ions in negative mode (m, z) [b]	identification	Structure type	Source	Reference
1	$C_6H_{14}N_4O_2$	2.15	175.1188 （-0.6）	173.1038 （-3.4）	175.1183$[M+H]^+$, 158.0917$[M+H-NH_3]^+$, 130.0962$[M+H-CH_3N_2]^+$, 116.0699[M+H-CH_3N_2-NH3]$^+$, 70.0645$[M+H-CH_3N_2-NH_3-HCOOH]^+$	173.1030$[M-H]^-$, 131.0819$[M-H-CH_3N_2]^-$,	L(+)-Arginine [a, c]	Organic acid	CH, HQ, JBX, DS, DZ, GC	
2	$C_{24}H_{42}O_{21}$	2.3	ND	665.2107 （-5.8）	ND	665.2090$[M-H]^-$, 485.1492$[M-H-Glc-H_2O]^-$, 443.1412$[M-H-Glc-C_2H_4O_2]^-$, 383.1145$[M-H-Glc-C_4H_8O_4]^-$, 341.1058$[M-H-2Glc]^-$, 179.0542	Stachyose [c]	Saccharides	HQ, DZ	
3	$C_4H_7NO_4$	2.3	134.0443 （-3.5）	132.0299 （-2.7）	ND	ND	Aspartic acid [a]	Organic acid	CH, JBX, DZ, GC	
4	$C_5H_9NO_4$	2.33	148.0599 （-2.5）	146.0456 （-1.8）	ND	ND	Glutamic acid [a]	Organic acid	CH, SJ, DZ, GC	
5	$C_5H_{12}O_5$	2.33	153.0755 （-1.5）	151.0607 （-3.3）	99.0434$[M+H-3H_2O]^+$, 69.0331$[M+H-3H_2O-CH_2O]^+$, 57.0329	151.0592$[M-H]^-$, 101.0237$[M-H-H_2O-CH_4O]^-$, 89.0227$[M-H-H_2O-CH_2O_2]^-$, 71.0128$[M-H-2H_2O-CH_2O_2]^-$, 59.0130	D-Arabitol or Ribitol [c]	Saccharides	CH	
6	$C_6H_{12}O_6$	2.34	ND	179.0556 （-2.4）	ND	179.0531$[M-H]^-$, 89.0226$[M-H-C_3H_7O_3]^-$, 71.0127$[M-H-C_3H_7O_3-H_2O]^-$, 59.0129$[M-H-C_4H_9O_4]^-$,	D-Tagatose or D-Glucose	Saccharides	CH, HQ, DS, SJ, DZ, GC	[1]
7	$C_6H_{12}O_7$	2.34	ND	195.0505 （-2.6）	ND	195.0499$[M-H]^-$, 129.0191$[M-H-HCOOH-CH_2O]^-$,	Galactonic acid [c]	Glycosides	CH, HQ, DS, DZ, GC	

No.	formula	t_R, min	$[M+H]^+$ (error, ppm)	$[M-H]^-$ (error, ppm)	Major fragment ions in positive mode (m, z) [b]	Major fragment ions in negative mode (m, z) [b]	identification	Structure type	Source	Reference
						75.0086$[M-H-HCOOH-CH_2O-3H_2O]^-$,				
8	$C_5H_{11}NO_2$	2.36	118.0857 （-4.7）	ND	ND	ND	Valine	Organic acid	CH, HQ, DS, DZ, GC	[2]
9	$C_6H_{13}NO_5$	2.36	180.8650 （-1.0）	ND	180.0872$[M+H]^+$, 162.0747$[M+H-H_2O]^+$, 144.0642$[M+H-2H_2O]^+$, 84.0434$[M+H-2H_2O-C_2H_4O_2]^+$, 72.0436$[M+H-2H_2O-C_3H_5O_2]^+$,	ND	Glucosamine Hydrochloride [c]	Saccharides	HQ, DS, DZ, GC	
10	$C_5H_9NO_2$	2.40	116.0700 （-4.9）	ND	116.0700$[M+H]^+$, 70.0646$[M+H-HCOOH]^+$,	ND	Proline [a, c]	Organic acid	HQ, JBX, DS, SJ, DZ, GC	
11	$C_{12}H_{22}O_{11}$	2.40	343.1234 （-0.2）	341.1071 （-5.3）	ND	341.1058$[M-H]^-$, 179.0555$[M-H-C_6H_{11}O_5]^-$, 161.0462$[M-H-C_6H_{11}O_5-H_2O]^-$, 89.0243$[M-H-C_2H_6O_2]^-$,	Sucrose [c]	Saccharides	HQ, DS, SJ, DZ, GC	
12	$C_6H_6O_3$	2.40	127.0382 （-2.9）	ND	127.0388$[M+H]^+$, 109.0282$[M+H-H_2O]^+$, 81.0329$[M+H-H_2O-CO]^+$, 69.0331$[M+H-C_2HO_2]^+$,	ND	5-Hydroxymethylfurfural [c]	Others	CH, JBX, DS, SJ, DZ, GC	
13	$C_7H_{12}O_6$	2.44	ND	191.0194 （-3.5）	ND	191.0546$[M-H]^-$, 127.0383$[M-H-H_2O-HCOOH]^-$, 85.0285	Quinic acid [c]	Phenolic acids	CH, DS	
14	$C_5H_5N_5$	2.47	136.0612 （-3.9）	ND	136.0613$[M+H]^+$, 119.0349$[M+H-NH_3]^+$,	ND	Adenine [c]	Others	CH, HQ, JBX, GC	
15	$C_4H_6O_5$	2.59	ND	133.0141 （-1.4）	ND	133.0136$[M-H]^-$, 115.0030$[M-H-H_2O]^-$, 71.0132$[M-H-H_2O-CO_2]^-$,	Malic acid [a, c]	Organic acid	CH, HQ, JBX, DS, SJ, DZ	
16	$C_4H_4O_4$	2.6	ND	115.0032 （-4）	ND	114.9981$[M-H]^-$, 71.0136$[M-H-CO_2]^-$,	Fumaric acid [c]	Organic acid	HQ, DS, DZ	

No.	formula	t_R, min	$[M+H]^+$ (error, ppm)	$[M-H]^-$ (error, ppm)	Major fragment ions in positive mode (m, z) [b]	Major fragment ions in negative mode (m, z) [b]	identification	Structure type	Source	Reference
17	$C_6H_8O_7$	3.23	193.0336 (-3.3)	191.0193 (-0.8)	139.0020$[M+H-3H_2O]^+$, 129.0173$[M+H-H_2O-HCOOH]^+$, 111.0073$[M+H-2H_2O-HCOOH]^+$, 68.9967	191.0195$[M-H]^-$, 111.0087$[M-H-2H_2O-CO_2]^-$, 87.0090$[M-H-2H_2O-2CO_2]^-$,	Citric acid [a,c]	Organic acid	CH, HQ, DS, SJ, DZ, GC	
18	$C_9H_{12}N_2O_6$	3.34	ND	243.0611 (-1.7)	ND	243.0613$[M-H]^-$, 200.0529$[M-H-CO_2]^-$,	Uridine [c]	Glycosides	CH, HQ, JBX, DS, GC	
19	$C_{10}H_{13}N_5O_4$	3.52	268.1038 (-0.7)	ND	268.1043$[M+H]^+$, 136.0614$[M+H-C_5H_9O_4]^+$,	ND	Adenosine [c]	Glycosides	CH, HQ, DS, GC	[3]
20	$C_6H_{13}NO_2$	3.66	132.1014 (-2.3)	130.0863 (-4.6)	132.1017$[M+H]^+$, 86.0960$[M+H-HCOOH]^+$, 69.0695$[M+H-HCOOH-NH_3]^+$	130.0860$[M-H]^-$, 113.0243$[M-H-NH_3]^-$, 88.0401$[M-HCOOH-NH_3]^-$,	Isoleucine	Organic acid	JBX,DS	[3]
21	$C_4H_6O_4$	3.75	ND	117.0189 (-3.6)	ND	117.0177$[M-H]^-$, 99.0089$[M-H-H_2O]^-$, 73.0287$[M-H-CO_2]^-$,	Succinic acid [a, c]	Organic acid	CH, DS	
22	$C_{10}H_{13}N_5O_5$	3.81	284.0990 (0.1)	282.0836 (-2.7)	284.1239$[M+H]^+$, 152.0564$[M+H-C_5H_9O_4]^+$, 135.0292$[M+H-C_5H_9O_4-NH3]^+$, 88.0749$[M+H-C_5H_9O_4-NH_3-CH_2NO]^+$,	282.0829$[M-H]^-$, 150.0413$[M-H-C_5H_9O_4]^-$, 133.0143$[M-H-C_5H_9O_4-NH_3]^-$,	Guanosine [c]	Glycosides	CH, HQ, JBX, DS	
23	$C_9H_{11}NO_2$	5.37	166.0860 (-1.7)	164.0709 (-3.2)	166.0861$[M+H]^+$, 120.0799$[M+H-HCOOH]^+$, 103.0533$[M+H-HCOOH-NH_3]^+$,	164.8333$[M-H]^-$, 147.0429$[M-H-NH_3]^-$, 120.9367$[M-H-CO_2]^-$, 103.0563$[M-H-NH_3-CO_2]^-$,	Phenylalanine [a, c]	Organic acid	HQ, JBX	
24	$C_{14}H_{21}NO_4$	6.02	268.1540 (-0.3)	ND	268.1530$[M+H]^+$, 161.0589$[M+H-C_4H_{11}NO-H_2O]^+$, 88.0750	ND	Codonopsine	Alkaloids	DS	[4]

No.	formula	t_R, min	$[M+H]^+$ (error, ppm)	$[M-H]^-$ (error, ppm)	Major fragment ions in positive mode (m, z) [b]	Major fragment ions in negative mode (m, z) [b]	identification	Structure type	Source	Reference
25	$C_{16}H_{18}O_9$	7.78	355.1012 （-3.1）	353.0851 （-6.4）	163.0385$[M+H-C_7H_{11}O_6]^+$,<br>145.0265$[M+H-C_9H_7O_3-HCOOH]$<br>135.0401$[M+H-C_8H_{11}O_7]^+$,	353.0857$[M-H]^-$,<br>191.0550$[M-H-C_9H_7O_3]^-$,<br>179.0345$[M-H-C_7H_{11}O_5]^-$,<br>173.0461$[M-H-C_9H_7O_3-H_2O]^-$,<br>135.0439$[M-H--C_7H_{11}O_5-CHO_2]^-$,	Neochlorogenic acid [a]	Phenylpropanoids	CH	
26	$C_7H_6O_3$	9.44	ND	137.0238 （-3.8）	ND	137.0228$[M-H]^-$,<br>119.0157$[M-H-H_2O]^-$,	Protocatechuic aldehyde [c]	Phenolic acids	GC	
27	$C_9H_{10}O_3$	9.55	ND	165.0553 （-1.3）	ND	165.0560$[M-H]^-$,<br>121.0653$[M+COOH]^-$,<br>93.0352$[M+COOH-C_2H_4]^-$,<br>59.0130	Desaminotyrosine [c]	Phenylpropanoids	GC	
28	$C_{16}H_{18}O_9$	9.97	355.1022 （-0.3）	353.0855 （-6.6）	163.0376$[M+H-C_7H_{11}O_6]^+$,<br>145.0280$[M+H-C_9H_7O_3-HCOOH]$<br>135.0429$[M+H-C_8H_{11}O_7]^+$,	353.0860$[M-H]^-$,<br>191.0549$[M-H-C_9H_7O_3]^-$,	Chlorogenic acid[a, c]	Phenylpropanoids	CH，HQ	
29	$C_{21}H_{22}O_9$	10.34	419.1338 （0.2）	ND	257.0794$[M+H-Glc]^+$,	ND	Isomer of liquiritin	Flavonoids	GC	[1]
30	$C_{27}H_{30}O_{15}$	11.4	595.1634 （-1.3）	593.1482 （-4.8）	595.1641,<br>577.1126$[M+H-H_2O]^+$,<br>559.1375$[M+H-2H_2O]^+$,<br>457.1091$[M+H-H_2O-C_4H_8O_4]^+$,<br>427.1029$[M+H-H_2O-C_5H_{10}O_5]^+$,<br>325.0767$[M+H-C_5H_{10}O_5-C_4H_8O_4]^+$	593.1483,<br>473.1060$[M-H-C_4H_8O_4]^-$,<br>353.0645$[M-H-2C_4H_8O_4]^-$	Vicenin-2 (Apigenin-6,8-di-*C*-glucoside) [b]	Flavonoids	HQ,	[5]
31	$C_9H_8O_4$	11.45	ND	179.0344 （-3.2）	ND	179.0342$[M-H]^-$,<br>135.0449$[M-H-CO_2]^-$,	Caffeic acid [a]	Phenolic acids	CH, GC，HQ	

No.	formula	t_R, min	$[M+H]^+$ (error, ppm)	$[M-H]^-$ (error, ppm)	Major fragment ions in positive mode (m, z) [b]	Major fragment ions in negative mode (m, z) [b]	identification	Structure type	Source	Reference
32	$C_{26}H_{28}O_{14}$	12.67	565.155 （-0.4）	563.1374 （-5.6）	565.1524$[M+H]^+$, 547.1394$[M+H-H_2O]^+$, 529.1295$[M+H-2H_2O]^+$ 511.1207$[M+H-3H_2O]^+$, 481.1100$[M+H-3H_2O-2CH_2O]^+$, 427.1004$[M+H-H_2O-C_4H_8O_4]^+$, 409.0909$[M+H-2H_2O-C_4H_8O_4]^+$, 379.0801$[M+H-2H_2O-C_4H_8O_4-2CH_2O]^+$,	563.1354$[M-H]^-$, 473.1079$[M-H-C_3H_6O_3]^-$ 443.0963$[M-H-C_4H_8O_4]^-$, 383.0732$[M-H-C_4H_8O_4-C_2H_4O_2]^-$, 353.0662$[M-H-C_3H_6O_3-C_4H_8O_4]^-$,	Schaftoside [a, c]	Flavonoids	HQ, GC	[6]
33	$C_{21}H_{20}O_9$	12.97	417.1167 (-3.1)	ND	417.1190 255.0655$[M+H-Glc]^+$	ND	Sophoraflavone B	Flavonoids	GC	[6]
34	$C_{26}H_{28}O_{14}$	13.17	565.1542 （-1.7）	563.1366 （-5.8）	565.1578$[M+H]^+$, 547.1413$[M+H-H_2O]^+$, 529.1316$[M+H-2H_2O]^+$, 481.1199$[M+H-3H_2O-CH_2O]^+$, 457.1180$[M+H-H_2O-C_3H_6O_3]^+$, 427.1149$[M+H-H_2O-C_4H_8O_4]^+$, 397.0939$[M+H-H_2O-C_4H_8O_4-CH_2O]^+$, 325.0721$[M+H-2C_4H_8O_4]^+$,	563.1358$[M-H]^-$, 473.1205$[M-H-C_3H_6O_3]^-$, 443.0925$[M-H-C_4H_8O_4]^-$, 383.0741[M-H-C4H8O4-$C_2H_4O_2]^-$, 353.0647$[M-H-C_3H_6O_3-C_4H_8O_4]^-$,	Isoschaftoside [a, c]	Flavonoids	HQ, GC	
35	$C_{27}H_{30}O_{14}$	13.35	ND	577.1527 （-6.1）	ND	577.1513$[M-H]^-$, 457.1140$[M-H- C_4H_8O_4]^-$, 337.0685$[M-H- 2C_4H_8O_4]^-$	Chrysin-6,8-di-C-glucoside	Flavonoids	HQ	[7]

No.	formula	t_R, min	$[M+H]^+$ (error, ppm)	$[M-H]^-$ (error, ppm)	Major fragment ions in positive mode (m, z) [b]	Major fragment ions in negative mode (m, z) [b]	identification	Structure type	Source	Reference
36	$C_{27}H_{32}O_{14}$	13.75	581.1846 （-0.1）	ND	581.1871$[M+H]^+$, 419.1337$[M+H-Glc]^+$, 257.0808$[M+H-2Glc]^+$,	ND	Glucoliquiritin	Flavonoids	GC	[6,8]
37	$C_{27}H_{30}O_{14}$	14.08	579.1700 （-1.5）	577.1530 （-5.7）	579.1665$[M+H]^+$, 561.1592$[M+H-H_2O]^+$, 543.1511$[M+H-2H_2O]^+$, 423.1077$[M+H-2H_2O-C_7H_5O_2]^+$,	577.1510$[M-H]^-$, 457.1161$[M-H- C_4H_8O_4]^-$, 383.0722$[M-H- C_4H_8O_4-C_3H_6O_2]^-$	Isoviolanthin	Flavonoids	GC	[6,9]
38	$C_{22}H_{22}O_{10}$	14.36	447.1287 （0.4）	ND	447.1288$[M+H]^+$ 285.0752$[M+H-Glc]^+$, 270.0612$[M+H-Glc-CH_3]^+$,	ND	Calycosin-7-o-glucoside [a, c]	Flavonoids	GC	
39	$C_{27}H_{30}O_{16}$	14.6	611.1593 （-0.7）	609.1026 （-5.8）	611.1654$[M+H]^+$, 465.1046$[M+H-C_6H_{11}O_4]^+$, 303.0499$[M+H-C_{12}H_{21}O_9]^+$,	609.1435$[M-H]^-$, 300.0262$[M-H-C_{12}H_{21}O_9]^-$,	Rutin[a, c]	Flavonoids	CH, DZ, GC	
40	$C_{21}H_{22}O_9$	14.62	419.1329 （-1.9）	417.1158 （-7.4）	257.0809$[M+H-Glc]^+$, 137.0229$[M+H-Glc-C_7H_5O_2]^+$,	417.1188$[M-H]^-$, 255.0649$[M-H-Glc]^-$, 135.0069$[M-H-Glc-C_7H_5O_2]^-$, 119.0497	Neoliquiritin	Flavonoids	GC	[10-12]
41	$C_{15}H_{12}O_4$	14.72	257.0806 （-0.7）	ND	257.0815$[M+H]^+$, 147.0429$[M+H-C_6H_5O_2]^+$, 137.0224$[M+H-C_8H_7O]^+$,	ND	Isomer of isoliquiritigenin [c]	Flavonoids	GC	
42	$C_{26}H_{30}O_{13}$	14.74	551.1744 （-2.8）	549.1571 （-7）	257.0807$[M+H-Api-Glc]^+$,	549.1563$[M-H]^-$, 255.0659$[M-H-Api-Glc]^-$,	Liquiritin apioside [a]	Flavonoids	GC	
43	$C_{15}H_{12}O_4$	15.00	257.0805 （-0.8）	ND	257.0807$[M+H]^+$ 147.0432$[M+H-C_6H_5O_2]^+$, 137.0228$[M+H-C_8H_7O]^+$,	ND	Isomer of liquiritigenin [c]	Flavonoids	GC	

No.	formula	t_R, min	$[M+H]^+$ (error, ppm)	$[M-H]^-$ (error, ppm)	Major fragment ions in positive mode (m, z) [b]	Major fragment ions in negative mode (m, z) [b]	identification	Structure type	Source	Reference
44	$C_{21}H_{22}O_9$	15.01	419.1321 (-3.0)	417.1160 (-6.7)	257.0801[M+H-Glc]+, 137.0218[M+H-Glc-C_8H_7O]+,	417.1164[M-H]-, 255.0657[M-H-Glc]-, 135.0086[M-H-Glc-C_8H_7O]-,	Liquiritin [a, c]	Flavonoids	GC	
45	$C_{29}H_{36}O_{15}$	15.02	ND	623.1947 (-5.6)	ND	623.1940[M-H]-, 461.1611[M-H-Glc]-, 161.0234	Acteoside [c]	Phenylpropanoids	HQ, GC	
46	$C_{21}H_{20}O_{12}$	15.14	465.1018 (-2.0)	463.0852 (-6.5)	465.0951[M+H]+, 289.0698[M+H-$C_6H_8O_6$]+, 169.0116[M+H-$C_6H_8O_6$-C_8H_8O]+,	417.1171[M+COOH]-, 255.0655[M-H-HCOOH-Glc]-, 135.0081[M-H-HCOOH-Glc-C_8H_7O]-,	Viscidulin I, 2′-O-glucoside	Flavonoids	HQ	[13]
47	$C_{21}H_{18}O_{12}$	15.6	463.0864 (-1.5)	461.0696 (-6.4)	463.0865[M+H]+, 287.0548[M+H-$C_6H_8O_6$]+,	461.0672[M-H]-, 285.0396[M-H-$C_6H_8O_6$]-,	Scutellarin [a]	Flavonoids	HQ	
48	$C_{21}H_{20}O_{12}$	15.88	465.1024 (-0.8)	463.0848 (-7.2)	465.1087[M+H]+, 289.0699[M+H-$C_6H_8O_6$]+, 169.0136[M+H-$C_6H_8O_6$-C_8H_8O]+,	463.0817[M-H]-, 287.0537[M-H-$C_6H_8O_6$]-, 269.0432[M-H-$C_6H_8O_6$-H_2O]-, 166.9969[M-H-$C_6H_8O_6$-C_8H_8O]-,	Carthamidin-7-O-glucuronide	Flavonoids	HQ	[14]
49	$C_{27}H_{30}O_{15}$	16.43	595.1634 (-0.6)	ND	595.1688[M+H]+, 433.1116[M+H-Glc]+, 271.0596[M+H-2Glc]+,	ND	Oroxin B [c]	Flavonoids	HQ	
50	$C_{23}H_{24}O_{12}$	16.56	493.1325 (-2.2)	ND	493.1290[M+H]+ 331.0795[M+H-Glc]+,	ND	5,2',6'-Trihydroxy-7,8-dimethoxyflavone 2′-O-glucoside	Flavonoids	HQ	[15]
51	$C_{21}H_{20}O_9$	16.8	417.1173 (-1.6)	415.1011 (-5.6)	417.1176, 399.1053[M+H-H_2O]+, 381.0955[M+H-2H_2O]+, 297.0748[M+H-$C_4H_8O_4$]+, 279.0661[M+H-H_2O-$C_4H_8O_4$]+	415.1016, 295.0607[M-H-$C_4H_8O_4$]-, 267.0662[M-H-$C_4H_8O_4$-CO]-	Chrysin 8-C-glucoside [c]	Flavonoids	HQ	[5,16]

No.	formula	t_R, min	$[M+H]^+$ (error, ppm)	$[M-H]^-$ (error, ppm)	Major fragment ions in positive mode (m, z) [b]	Major fragment ions in negative mode (m, z) [b]	identification	Structure type	Source	Reference
52	$C_{21}H_{22}O_{10}$	17.43	ND	433.1108 (-4.7)	ND	433.1113<br>271.0600[M-H-Glc]- ,<br>151.0020[M-H-Glc-$C_7H_4O_2$]-	5-Hydroxylliquiritin	Flavonoids	GC	[17]
53	$C_{20}H_{28}O_8$	18.01	ND	441.1740 （-3.5）	ND	441.1735$[M+COOH]^-$,<br>215.1064$[M-H-C_6H_{12}O_6]^-$,<br>179.0549$[M-H-C_{14}H_{16}O_2]^-$,<br>143.0719$[C_7H_{11}O_3]^-$<br>89.0217$[M-H- C_6H_{11}O_5-C_7H_{12}O_3]^-$,	Lobetyolin [a]	Glycosides	DS	[4]
54	$C_{22}H_{20}O_{12}$	18.18	477.1007 （-2.5）	475.0847 （-7.3）	477.0995$[M+H]^+$,<br>301.0694$[M+H- C_6H_8O_6]^+$,<br>286.0463$[M+H-C_6H_8O_6-CH_3]^+$	475.0829$[M-H]^-$,<br>299.0550$[M-H- C_6H_8O_6]^-$,<br>284.0326$[M-H- C_6H_8O_6-CH_3]^-$,<br>175.0257$[M-H-C_{16}H_{12}O_6]^-$	5,7,2'-Trihydroxy-6-methoxyflavone-7-*O*-glucuronide	Flavonoids	HQ	[5]
55	$C_{21}H_{22}O_9$	18.48	419.1329 （-1.7）	ND	419.1469$[M+H]^+$,<br>257.0811$[M+H-Glc]^+$,<br>239.0649$[M+H-Glc-H_2O]^+$,<br>149.0234, 137.0219	ND	Isomer of liquiritin	Flavonoids	GC	[18]
56	$C_{26}H_{30}O_{13}$	18.49	551.1738 （-3.9）	549.1575 （-6.4）	419.1327$[M+H-Api]^+$,<br>257.0807$[M+H-Api-Glc]^+$,<br>137.0227$[M+H-Api-Glc-C_7H_5O_2]^+$,	549.1570$[M-H]^-$,<br>255.0642$[M-H-Api-Glc]^-$,<br>135.0081$[M-H-Api-Glc-C_7H_5O_2]^-$,	Isoliquiritin apioside	Flavonoids	GC	[10]
57	$C_{26}H_{30}O_{13}$	18.84	551.1732 （-3.2）	549.1577 （-6.7）	551.1738$[M+H]^+$,<br>419.1325$[M+H-Api]^+$,<br>257.0796$[M+H-Api-Glc]^+$,<br>137.0210$[M+H-Api-Glc-C_7H_5O_2]^+$,	549.1563$[M-H]^-$,<br>429.1024$[M-H-C_7H_5O_2]^-$,<br>255.0637$[M-H-Api-Glc]^-$,	Liguiritigenin-7-O-D-apiosyl-4’-O-D-glucoside	Flavonoids	GC	[10]
58	$C_{22}H_{22}O_9$	19.06	431.1324 （-3.0）		269.0803$[M+H-Glc]^+$,	ND	Ononin [c]	Flavonoids	GC	[18]

No.	formula	t_R, min	$[M+H]^+$ (error, ppm)	$[M-H]^-$ (error, ppm)	Major fragment ions in positive mode (m, z) [b]	Major fragment ions in negative mode (m, z) [b]	identification	Structure type	Source	Reference
59	$C_{15}H_{10}O_6$	19.13	287.0542 （-1.2）	285.0384 （-5.3）	287.0540$[M+H]^+$, 269.0432$[M+H-H_2O]^+$, 241.0489$[M+H-H_2O-CO]^+$, 153.0162$[M+H-C_8H_6O_2]^+$, 139.0014$[M+H-C_9H_6O_2]^+$,	285.0391$[M-H]^-$, 151.0024$[M-H-C_8H_6O_2]^-$, 107.0142$[M-H-C_8H_6O_2-CO_2]^-$,	Scutellarein	Flavonoids	HQ	[5]
60	$C_{21}H_{20}O_9$	19.17	417.1160 (-0.5)	415.1030 (-1.2)	417.1166, 381.0976$[M+H-2H_2O]^+$, 351.0848$[M+H-2H_2O-CH_2O]^+$, 321.0786$[M+H-2H_2O-C_2H_4O_2]^+$, 297.0746$[M+H-C_4H_8O_4]^+$, 267.0635$[M+H-C_5H_{10}O_5]^+$	415.1003, 325.0710$[M-H-C_3H_6O_3]^-$, 295.0572$[M-H-C_4H_8O_4]^-$, 267.0645$[M-H-C_4H_8O_4-CO]^-$,	Chrysin 6-C-glucoside	Flavonoids	HQ	[5,16]
61	$C_{21}H_{18}O_{12}$	19.24	463.0854 （0.6）	ND	287.0532$[M+H- C_6H_8O_6]^+$, 269.0432$[M+H- C_6H_8O_6 -H_2O]^+$, 241.0468	ND	Luteolin-7-O-β-D-glucuronide [c]	Flavonoids	HQ	[19]
62	$C_{21}H_{18}O_{11}$	19.26	447.0904 （-3.5）	445.0745 （-6.8）	447.0885$[M+H]^+$, 271.0583$[M+H-C_6H_8O_6]^+$,	445.0727$[M-H]^-$, 269.0439$[M-H-C_6H_8O_6]^-$,	Baicalin [a]	Flavonoids	HQ, GC	
63	$C_{21}H_{22}O_9$	19.28	419.1324 （-3.1）	417.1166 （-5.9）	419.1321$[M+H]^+$, 257.0797$[M+H-Glc]^+$, 137.0224	417.1147$[M-H]^-$, 297.0711$[M-H-C_4H_8O_4]^-$, 255.0650$[M-H-Glc]^-$, 148.0163, 135.0082	Isoliquiritin [a]	Flavonoids	GC	
64	$C_{42}H_{68}O14$	19.47	ND	795.4515 （-2.7）	ND	841.4527$[M+COOH]^-$, 795.4515$[M-H]^-$, 633.4030$[M-H-Glc]^-$,	21β-Hydroxysaikosaponin b2	Triterpenoid saponin	CH	[20]
65	$C_{21}H_{22}O_9$	19.69	419.1322 （-3.5）	417.1159 （-7.7）	419.1338$[M+H]^+$, 257.0800$[M+H-Glc]^+$, 147.0424, 137.0234	417.1153$[M-H]^-$, 255.0656$[M-H-Glc]^-$, 135.0089, 119.0498	Neoisoliquiritin	Flavonoids	GC	[11]

No.	formula	t_R, min	$[M+H]^+$ (error, ppm)	$[M-H]^-$ (error, ppm)	Major fragment ions in positive mode (m, z) [b]	Major fragment ions in negative mode (m, z) [b]	identification	Structure type	Source	Reference
66	$C_{35}H_{36}O_{15}$	19.77	697.2121 （-0.9）	695.1939 （-2.9）	697.1801<br>257.0770[$M+H-C_{20}H_{25}O_{11}$]$^-$<br>167.0331	695.1927<br>549.1573[$M-H-C_9H_7O_2$]$^-$,<br>255.0639[$M-H-C_{20}H_{25}O_{11}$]$^-$	Licorice glycoside B	Flavonoids	GC	[6]
67	$C_{15}H_{10}O_4$	20.24	255.0646（-2.3）	253.0498 （-3.4）	255.0642[$M+H$]$^+$<br>137.0227[$M+H-C_8H_6O$]$^+$	253.0495[$M-H$]$^-$,<br>135.0083[$M-H-C_8H_6O$]$^-$,<br>117.0345[$M-H-C_7H_4O_3$]$^-$,	4′,7-Dihydroxyflavone	Flavonoids	GC	[21]
68	$C_{15}H_{12}O_5$	20.25	273.0747（-2.4）	ND	273.0745[$M+H$]$^+$,<br>169.0115[$M+H-C_8H_8$]$^+$,<br>131.0472[$M+H-C_6H_4O_4$]$^+$,	ND	Dihydrobaicalein	Flavonoids	HQ	[22]
69	$C_{21}H_{20}O_{11}$	20.26	449.1057 （-4.7）	447.0898 （-7.9）	273.0748[$M+H-C_6H_8O_6$]$^+$<br>169.0124	447.0891[$M-H$]$^-$,<br>271.0597[$M-H-C_6H_8O_6$]$^-$,	Naringenin-7-O-Glu acid	Flavonoids	HQ	[23]
70	$C_{31}H_{40}O_{15}$	20.4	ND	651.2254 （-6.2）	ND	651.2247[$M-H$]$^-$,<br>475.1895[$M-H-C_6H_8O_6$]$^-$,<br>175.0422	Epimeredinoside A [c]	Glycosides	HQ	
71	$C_{21}H_{20}O_{11}$	20.62	449.1043 （-5.0）	447.0898 （-7.1）	449.0977[$M+H$]$^+$,<br>273.0736[$M+H-C_6H_8O_6-C_8H_8$]$^+$,<br>169.0121	447.0895[$M-H$]$^-$,<br>271.0606[$M-H-C_6H_8O_6$]$^-$,<br>243.0661[$M-H-C_6H_8O_6-CO$]$^-$,	5,6,7-Trihydroxy flavanone (Dihydroxybaicalein)-7-O-glucuronide	Flavonoids	HQ	[5,24]
72	$C_{21}H_{18}O_{11}$	20.75	447.0905 （-3.2）	445.0745 （-6.3）	447.0912[$M+H$]$^+$,<br>271.0597[$M+H-C_6H_8O_6$]$^+$,	445.0738[$M-H$]$^-$,<br>269.0444[$M-H-C_6H_8O_6$]$^-$,	Baicalein 6-O-glucuronide	Flavonoids	HQ	[23]
73	$C_{22}H_{20}O_{12}$	20.92	477.1007 （-4.2）	475.0846 （-4.9）	477.1019[$M+H$]$^+$,<br>301.0696[$M+H-C_6H_8O_6$]$^+$,<br>286.0462[$M+H-C_6H_8O_6-CH_3$]$^+$	475.0858[$M-H$]$^-$,<br>299.0560[$M-H-C_6H_8O_6$]$^-$,<br>284.0311[$M-H-C_6H_8O_6-CH_3$]$^-$,<br>175.0241[$M-H-C_{16}H_{12}O_6$]$^-$	5,7,8-Trihydroxy-6-methoxyflavone-7-O-glucuronide	Flavonoids	HQ	[5,16]

No.	formula	t_R, min	[M+H]+ (error, ppm)	[M-H]- (error, ppm)	Major fragment ions in positive mode (m, z) [b]	Major fragment ions in negative mode (m, z) [b]	identification	Structure type	Source	Reference
74	$C_{22}H_{22}O_{10}$	21.06	447.0904 (-3)	ND	447.0913[M+H]+,<br>285.0745[M+H-Glc]+,<br>271.0596[M+H-$C_6H_8O_6$]+,	ND	Wogonin 7-glucoside	Flavonoids	HQ	[5,24]
75	$C_{31}H_{40}O_{15}$	21.2	ND	651.2257 (-5.8)	ND	651.2244[M-H]-,<br>351.0559[M-H-$C_9H_{11}O_2$-$C_9H_9O_2$]-,<br>175.0394[M-H-$C_9H_{11}O_2$-$C_{12}H_{20}O_{10}$]-	Isomartynoside	Glycosides	HQ	[5]
76	$C_{15}H_{12}O_4$	21.31	257.0808 (-0.2)	255.0656 (-2.8)	257.0801[M+H]+,<br>147.0425[M+H-$C_6H_5O_2$]+,<br>137.0218[M+H-C_8H_7O]+,	255.0669[M-H]-,<br>219.8440[M-H-2H_2O]-,<br>201.8325[M-H-3H_2O]-,<br>135.0082[M-H-C_8H_7O]-,<br>119.0498[M-H-$C_7H_5O_3$]-,<br>91.0199[M-H-$C_7H_5O_3$-C_2H_4]-,	Liquiritigenin [a, c]	Flavonoids	GC	[15]
77	$C_{22}H_{20}O_{11}$	21.37	461.1064 (-3.2)	459.0904 (-6.3)	461.1058[M+H]+,<br>285.0745[M+H-$C_6H_8O_6$]+,<br>270.0518[M+H-$C_6H_8O_6$-CH_3]+,	459.0884[M-H]-,<br>283.0599[M-H-$C_6H_8O_6$]-,<br>268.0369[M-H-$C_6H_8O_6$-CH_3]-,<br>175.0240, 113.0234	Oroxyloside (Oroxylin A-7-O-glucuronide)	Flavonoids	HQ	[23]
78	$C_{42}H_{64}O_{16}$	21.42	825.4241 (-0.2)	823.4093 (-3.5)	825.4147<br>649.3935<br>455.3506[M+H-$C_{12}H_{16}O_{13}$]+,	823.4092	Licorice saponin J2	Triterpenoid saponin	GC	[5]
79	$C_{22}H_{20}O_{12}$	21.64	477.1010 (-3.7)	475.0848 (-6.4)	477.1020[M+H]+,<br>301.0701[M+H-$C_6H_8O_6$]+,<br>286.0472[M+H-$C_6H_8O_6$-CH_3]	475.0853[M-H]-,<br>299.0554[M-H-$C_6H_8O_6$]-,<br>284.0313[M-H-$C_6H_8O_6$-CH_3]-,	5,6,7-Trihydroxy-8-methoxyflavone-7-O-glucuronide	Flavonoids	HQ	[6]

No.	formula	t_R, min	$[M+H]^+$ (error, ppm)	$[M-H]^-$ (error, ppm)	Major fragment ions in positive mode (m, z) [b]	Major fragment ions in negative mode (m, z) [b]	identification	Structure type	Source	Reference
80	$C_{42}H_{70}O_{14}$	21.94	799.4790 （-6）	797.4646 （-2.9）	ND	843.4675$[M+COOH]^-$, 797.4646$[M-H]^-$, 635.4054$[M-H-Glc]^-$	Hydroxysaikosaponin A	Triterpenoid saponin	CH	[5,24]
81	$C_{16}H_{12}O_5$	22.31	285.0746 （-2.6）	283.0589 （-1）	285.0750$[M+H]^+$, 270.0512$[M+H-CH_3]^+$,	283.0587$[M-H]^-$, 268.0364$[M-H-CH_3]^-$, 163.0043$[M-H-CH_3-C_7H_5O]^-$,	Isomer of wogonin	Flavonoids	HQ	[20]
82	$C_{22}H_{20}O_{11}$	22.32	461.1062 （-3.1）	459.0900 （-6.2）	461.1048$[M+H]^+$, 285.0744$[M+H-C_6H_8O_6]^+$, 270.0514$[M+H-C_6H_8O_6-CH_3]^+$,	459.0900$[M-H]^-$, 283.0598$[M-H-C_6H_8O_6]^-$, 268.0363$[M-H-C_6H_8O_6-CH_3]^-$, 175.0249 113.0241	Wogonoside [a]	Flavonoids	HQ	
83	$C_{15}H_{10}O_7$	22.4	303.0502 （0.8）	301.0354 （0）	ND	301.0299$[M-H]^-$, 178.9955$[M-H-C_6H_4O_3]^-$, 151.0064$[M-H-C_8H_6O_3]^-$,	Quercetin [a]	Flavonoids	CH	
84	$C_{48}H_{72}O_{21}$	22.69	985.4640 （0.1）	983.4446 （-4.4）	985.4612 809.4258$[M+H-C_6H_8O_6]^+$, 647.3756$[M+H-C_6H_8O_6-C_6H_{10}O_5]^+$, 615.3866$[M+H-C_6H_8O_6-C_6H_8O_7]^+$ 453.3352$[M+H-C_6H_8O_6-C_6H_{10}O_5-C_6H_8O_7]^+$	983.4468	Licorice saponin A3	Triterpenoid saponin	GC	[6]
85	$C_{42}H_{62}O_{17}$	24.21	839.4021 （-4.6）	837.3871 （-4.7）	839.4033$[M+H]^+$, 663.3747$[M+H-Glc]^+$, 469.3297$[M+H-2Glc-H_2O]^+$,	837.3855$[M-H]^-$,	Uralsaponin N	Triterpenoid saponin	GC	[6]

No.	formula	t_R, min	$[M+H]^+$ (error, ppm)	$[M-H]^-$ (error, ppm)	Major fragment ions in positive mode (m, z) [b]	Major fragment ions in negative mode (m, z) [b]	identification	Structure type	Source	Reference
86	$C_{16}H_{12}O_6$	24.71	301.0699（-0.4）	299.0547（-4.6）	301.0713<br>286.0472$[M+H-CH_3]^+$,<br>168.0128$[M+H-CH_3-C_8H_6O]^+$	299.0550<br>284.0303$[M-H-CH_3]^-$,<br>136.9875	Trihydroxy-methoxyfavone	Flavonoids	HQ	[12]
87	$C_{15}H_{10}O_5$	25.54	271.0594（-2.5）	269.0444（-3）	271.0599$[M+H]^+$,<br>169.0133$[M+H-C_8H_6]^+$	269.0446$[M-H]^-$,<br>225.0537$[M-H-CO_2]^-$,<br>197.0606$[M-H-CO_2-CO]^-$,<br>171.0433	Norwogonin	Flavonoids	HQ	[5,15]
88	$C_{48}H_{78}O_{17}$	25.61	927.5252（-1.7）	925.5058（-3.2）	927.5343$[M+H]^+$,<br>747.4375$[M+H-C_6H_{11}O_6]^+$,<br>603.4330$[M+H-C_6H_{11}O_5-C_6H_{11}O_4-CH_3]^+$,<br>421.3416$[M+H-2C_6H_{11}O_5-C_6H_9O_5-H_2O]^+$,<br>309.1190	971.5080$[M+COOH]^-$,<br>925.5058$[M-H]^-$	Saikosaponin c [a]	Triterpenoid saponin	CH	[5]
89	$C_{16}H_{12}O_6$	25.7	301.0699（-2.6）	299.0544（-4.8）	301.0698$[M+H]^+$,<br>286.0459$[M+H-CH_3]^+$,<br>183.9985$[M+H-CH_3-C_8H_6]^+$,	299.0547$[M-H]^-$,<br>284.0312$[M-H-CH_3]^-$,<br>153.9900$[M-H-CH_3-C_9H_6O]^-$	5,7,8-Trihydroxy-6-methoxyflavone	Flavonoids	HQ	[25]
90	$C_{15}H_{10}O_5$	26.17	271.0595（-2.2）	269.0442（-3.6）	271.0592$[M+H]^+$<br>123.0068$[M+H-H_2O-C_9H_6O]^+$,	269.0435$[M-H]^-$,<br>169.0656$[M-H-C_8H_6]^-$,	Baicalein [a]	Flavonoids	HQ	[26]
91	$C_{42}H_{62}O_{17}$	26.2	839.4060（0.1）	837.3867（-5.1）	839.3984$[M+H]^+$,<br>663.3678$[M+H-Glc]^+$,<br>487.3388$[M+H-2Glc]^+$,<br>469.3286$[M+H-2Glc-H_2O]^+$,	837.3866$[M-H]^-$,	Yunganoside K2	Triterpenoid saponin	GC	[6]
92	$C_{48}H_{80}O_{17}$	26.44	929.5457（-1.2）	927.5261 (-4.2)	929.5431<br>767.4906$[M+H-Glc]^+$,	973.5311$[M+COOH]^-$<br>927.5261$[M-H]^-$	Saikosaponin f	Triterpenoid saponin	CH	[6]

No.	formula	t_R, min	$[M+H]^+$ (error, ppm)	$[M-H]^-$ (error, ppm)	Major fragment ions in positive mode (m, z) [b]	Major fragment ions in negative mode (m, z) [b]	identification	Structure type	Source	Reference
					471.1678$[M+H-Glc-C_6H_{11}O_4-C_5H_8O_5]^+$,<br>441.3737$[M+H-2C_6H_9O_6-C_6H_{11}O_4]^+$,<br>423.3608$[M+H-2Glc-C_6H_{11}O_4-2H_2O]^+$,					
93	$C_{42}H_{62}O_{17}$	26.99	837.3871 (-2.7)	837.3871 (-5.2)	839.4016$[M+H]^+$,<br>663.3718$[M+H-Glc]^+$,<br>487.3405$[M+H-2Glc]^+$,<br>469.3302$[M+H-2Glc-H_2O]^+$,	837.3869$[M-H]^-$,	Licorice saponin G	Triterpenoid saponin	GC	[25]
94	$C_{16}H_{12}O_6$	27.07	301.0709 (0.9)	299.0551 (-3.5)	301.0702<br>286.0478$[M+H-CH_3]^+$,<br>168.0050	299.0534<br>284.0319$[M-H-CH_3]^-$,<br>137.9952	Hispidulin	Flavonoids	HQ	[6,12]
95	$C_{42}H_{62}O_{17}$	27.37	839.4023 (-4.3)	837.3874 (-4.6)	839.4030$[M+H]^+$,<br>645.3597$[M+H-Glc-H_2O]^+$,<br>487.3383$[M+H-2Glc]^+$,<br>469.3294$[M+H-2Glc-H_2O]^+$,	837.3872$[M-H]^-$,	Licorice saponin G2	Triterpenoid saponin	GC	[27]
96	$C_{15}H_{12}O_4$	27.6	257.0805 (-1.3)	255.0655 (-2.9)	257.0806$[M+H]^+$,<br>147.0424$[M+H-C_6H_5O_2]^+$,<br>137.0219$[M+H-C_8H_7O]^+$,	255.0659$[M-H]^-$,<br>219.8439$[M-H-2H_2O]^-$,<br>201.8345$[M-H-3H_2O]^-$,<br>135.0078$[M-H-C_8H_7O]^-$,<br>119.0497$[M-H-C_7H_5O_3]^-$,<br>91.0179$[M-H-C_7H_5O_3-C_2H_4]^-$,	Isoliquiritigenin [a, c]	Flavonoids	GC	[6]
97	$C_{42}H_{62}O_{16}$	27.74	823.4069 (-4.8)	821.3922 (-5.3)	823.4046$[M+H]^+$,<br>647.3737$[M+H-C_6H_9O_6]^+$,<br>453.3321$[M+H-C_6H_9O_6-C_6H_8O_7]^+$,	821.3885$[M-H]^-$,	Glycyrrhizic acid [a]	Triterpenoid saponin	GC	

No.	formula	t_R, min	$[M+H]^+$ (error, ppm)	$[M-H]^-$ (error, ppm)	Major fragment ions in positive mode (m, z) [b]	Major fragment ions in negative mode (m, z) [b]	identification	Structure type	Source	Reference
98	$C_{16}H_{12}O_4$	27.88	269.0807 (-0.5)	267.0649 (0.8)	269.0802$[M+H]^+$,	267.0647$[M-H]^-$, 252.0428$[M-H-CH_3]^-$,	Formononetin [a,c]	Flavonoids	GC	
99	$C_{42}H_{68}O_{13}$	28.74	781.4694 (-5.0)	779.4536 (-6.5)	781.4638$[M+H]^+$, 745.4519$[M+H-2H_2O]^+$, 619.4267$[M+H-Glc]^+$, 455.3498$[M+H-Glc-H_2O-Fuc]^+$, 437.3397$[M+H-Glc-2H_2O-Fuc]^+$, 419.3331$[M+H-Glc-3H_2O-Fuc]^+$,	779.4585$[M-H]^-$, 617.4278$[M-H-Glc]^-$,	Saikosaponin a [a]	Triterpenoid saponin	CH	
100	$C_{42}H_{64}O_{15}$	28.77	809.4039 (-1.2)	807.4125 (-5)	809.4331 633.3988$[M+H-C_6H_9O_6]^+$, 457.3668$[M+H-C_6H_9O_6-C_6H_8O_6]^+$, 439.3576$[M+H-C_6H_9O_6-C_6H_8O_6-H_2O]^+$,	807.4097 351.0550	Licorice saponin B2	Triterpenoid saponin	GC	[6]
101	$C_{30}H_{46}O_3$	28.99	455.3501 (-4.1)	ND	455.3469$[M+H]^+$, 437.3337$[M+H-H_2O]^+$, 419.3270$[M+H-2H_2O]^+$, 407.3309$[M+H-H_2O-2CH_3]^+$,	ND	Betulonicacid [c]	Terpenoids	CH, DZ	[6,28,29]
102	$C_{42}H_{68}O_{13}$	29.01	781.4689 (-5.6)	779.4550 (-4.7)	763.4559$[M+H-H2O]^+$, 619.4174$[M+H-Glc]^+$, 455.3496$[M+H-Glc-H_2O-Fuc]^+$, 437.3399$[M+H-Glc-2H_2O-Fuc]^+$, 419.3257$[M+H-Glc-3H_2O-Fuc]^+$,	779.4516$[M-H]^-$, 617.4070$[M-H-Glc]^-$,	Saikosaponin b_2 [a]	Triterpenoid saponin	CH	[28,29]
103	$C_{42}H_{62}O_{16}$	29.06	823.4065 (-5.2)	821.3917 (-5.7)	823.4081$[M+H]^+$, 647.3743$[M+H-C_6H_9O_6]^+$, 453.3335$[M+H-C_6H_9O_6-C_6H_8O_7]^+$,	821.3874$[M-H]^-$,	Uralsaponin B	Triterpenoid saponin	GC	[6]

No.	formula	tR, min	[M+H]+ (error, ppm)	[M-H]- (error, ppm)	Major fragment ions in positive mode (m, z) b	Major fragment ions in negative mode (m, z) b	identification	Structure type	Source	Reference
104	$C_{21}H_{20}O_6$	29.41	369.1323（-2.6）	367.1162（-6.8）	369.1289$[M+H]^+$, 313.0713$[M+H-C_3H_4O]^+$, 271.0602$[M+H-2CH_2O-2H_2O]^+$	367.1171$[M-H]^-$, 309.0395$[M-H-2CH_2O]^-$	Curcumin c	Phenolic acids	SJ	[6]
105	$C_{48}H_{76}O_{19}$	29.48	957.5049（-0.5）	955.4845（-6.1）	957.5024 811.4426$[M+H-C_6H_{10}O_4]^+$, 599.3877$[M+H-C_6H_{10}O_4-C_6H_8O_6-2H_2O]^+$, 441.3682$[M+H-C_6H_{10}O_4-2C_6H_8O_6-2H_2O]^+$, 423.3613$[M+H-C_6H_{10}O_4-2C_6H_8O_6-3H_2O]^+$, 353.0702$[C_{12}H_{16}O_{12}]$	955.4848	Yunganoside A1	Triterpenoid saponin	GC	[6]
106	$C_{30}H_{46}O_3$	30.14	455.3499（-4.6）	ND	455.3491$[M+H]^+$, 437.3347$[M+H-H_2O]^+$, 419.3246$[M+H-2H_2O]^+$,	ND	Isomer of Betulonic acid c	Terpenoids	CH	[6]
107	$C_{44}H_{70}O_{14}$	30.33	823.4813（-3.1）	821.4613（-4.8）	823.4805$[M+H]^+$, 647.3682 455.3512$[M+H-C_2H_2O\text{-Glc-Fuc-}H_2O]^+$, 437.3386$[M+H-C_2H_2O\text{-Glc-Fuc-}2H_2O]^+$,	867.4665$[M+COOH]^-$, 821.4613$[M-H]^-$, 779.4516$[M-H-C_2H_2O]^-$, 761.4461$[M-H-C_2H_2O-H_2O]^-$, 617.3947$[M-H-C_2H_2O\text{-Glc}]^-$	2″-O-Acetyl-Saikosaponin a	Triterpenoid saponin	CH	[20]
108	$C_{42}H_{64}O_{16}$	30.5	825.4259（-1）	823.4073（-5.8）	825.4260$[M+H]^+$, 455.3499$[M+H-2C_6H_8O_6-H_2O]^+$, 437.3416$[M+H-2C_6H_8O_6-2H_2O]^+$	823.4065 351.0539$[M-H\text{-2Glc}]^-$	Uralsaponin C	Triterpenoid saponin	GC	[20]

No.	formula	t_R, min	$[M+H]^+$ (error, ppm)	$[M-H]^-$ (error, ppm)	Major fragment ions in positive mode (m, z) [b]	Major fragment ions in negative mode (m, z) [b]	identification	Structure type	Source	Reference
109	$C_{30}H_{46}O_3$	30.58	455.3498 （4.8）	ND	455.3504$[M+H]^+$,<br>407.3294$[M+H-H_2O-2CH_3]^+$,	ND	Isomer of betulonic acid [c]	Terpenoids	CH	[25]
110	$C_{42}H_{68}O_{13}$	30.65	781.4696 （-4.6）	826.4630 （-9.6）	763.4532$[M+H-H_2O]^+$,<br>601.4086$[M+H-Glc-H2O]^+$,<br>455.3492$[M+H-Glc-H2O-Fuc]^+$,<br>437.3391$[M+H-Glc-2H2O-Fuc]^+$,<br>419.3282$[M+H-Glc-3H2O-Fuc]^+$,	826.4621$[M+COOH]^-$,<br>780.4575<br>618.4015$[M-Glc]^-$,	Saikosaponin b1 [a]	Triterpenoid saponin	CH	
111	$C_{44}H_{70}O_{14}$	30.75	823.4762 （-5.6）	821.4645 （-5.2）	823.4070$[M+H]^+$,<br>805.4739$[M+H-H_2O]^+$,<br>611.3492<br>455.3484$[M+H-C_2H_2O-Glc-Fuc-H_2O]^+$,<br>453.3362$[M+H-C_2H_2O-Glc-Fuc-2H_2O]^+$	867.4697$[M+COOH]^-$,<br>821.4645$[M-H]^-$,<br>779.4562$[M-H-C_2H_2O]^-$,<br>761.4430$[M-H-C_2H_2O-H_2O]^-$,	2″-O-Acetyl-Saikosaponin b2	Triterpenoid saponin	CH	[20]
112	$C_{16}H_{12}O_5$	31.14	285.0750 （-2.8）	283.0599 （-2.4）	285.0745$[M+H]^+$,<br>270.0511$[M+H-CH_3]^+$,	283.0602$[M-H]^-$,<br>268.0362$[M-H-CH_3]^-$,<br>163.0029$[M-H-CH_3-C_7H_5O]^-$,	Wogonin [a, c]	Flavonoids	HQ	[20]
113	$C_{36}H_{58}O_8$	31.31	ND	617.3953 (-4.3).	ND	663.4081$[M+COOH]^-$,<br>617.3593$[M-H]^-$,	Prosaikogenin F	Triterpenoid saponin	CH	[20]

No.	formula	t_R, min	$[M+H]^+$ (error, ppm)	$[M-H]^-$ (error, ppm)	Major fragment ions in positive mode (m, z) [b]	Major fragment ions in negative mode (m, z) [b]	identification	Structure type	Source	Reference
114	$C_{42}H_{62}O_{15}$	31.44	807.4110 （-6.4）	805.3977 （-4.4）	807.4176 631.3825$[M+H-C_6H_8O_6]^+$, 455.3489$[M+H-C_{12}H_{16}O_{12}]^+$, 437.3404$[M+H-C_{12}H_{17}O_{13}]^+$, 419.3287$[M+H-C_{12}H_{17}O_{13}-H_2O]^+$	805.3985 351.0542$[M-H-C_{30}H_{45}O_3]^-$	Licorice saponin C_2	Triterpenoid saponin	GC	[25]
115	$C_{17}H_{14}O_6$	31.47	315.0855 （-2.5）	ND	315.0843$[M+H]^+$, 300.0611$[M+H-CH_3]^+$, 285.0385$[M+H-2CH_3]^+$, 282.0518$[M+H-CH_3-H_2O]^+$, 182.9911$[M+H-C_9H_8O]^+$, 154.9966{$M+H-C_{10}H_8O_2]^+$,	ND	5,7-Dihydroxy-6,8-dimethoxyfavone	Flavonoids	HQ	[6]
116	$C_{15}H_{10}O_4$	31.59	255.0649 （-1.3）	253.0497 （-3.7）	255.0645$[M+H]^+$, 153.0159	253.0494$[M-H]^-$, 209.0600$[M-H-CO_2]^-$, 143.0500	Chrysin [a]	Flavonoids	HQ, GC	[5,24]
117	$C_{19}H_{18}O_8$	31.71	375.1060 （-3.9）	373.0903 （-6.9）	375.0809$[M+H]^+$, 345.0586$[M+H-2CH_3]^+$, 327.0477$[M+H-2CH_3-H_2O]^+$, 197.0074$[M+H-C_{10}H_{10}O_3]^+$,	373.0906$[M-H]^-$, 358.0668$[M-H-CH_3]^-$, 343.0435$[M-H-2CH_3]^-$, 328.0199$[M-H-3CH_3]^-$, 300.0228$[M-H-3CH_3-CO]^-$, 151.9756$[M-H-C_{11}H_{11}O_5]^-$,	Skullcapflavone II (Neobaicalein)	Flavonoids	HQ	[5]
118	$C_{16}H_{12}O_5$	32.24	285.0750 （-2.7）	283.0601 （-3.9）	285.0752$[M+H]^+$, 270.0517$[M+H-CH_3]^+$, 168.0048	283.0588$[M-H]^-$, 268.0365$[M-H-CH_3]^-$,	Oroxylin A	Flavonoids	HQ	[5]
119	$C_{44}H_{70}O_{14}$	32.35	823.4798 （-4）	821.3890 （-4.9）	823.4716$[M+H]^+$, 805.4647$[M+H-H_2O]^+$, 455.3489$[M+H-C_2H_2O-Glc-Fuc-H_2O]^+$, 437.3394$[M+H-C_2H_2O-Glc-Fuc-2H_2O]^+$,	821.4589$[M-H]^-$, 779.4503$[M-H-C_2H_2O]^-$, 761.4277$[M-H-C_2H_2O-H_2O]^-$, 617.4114$[M-H-C_2H_2O-Glc]^-$	3″-O-Acetyl-Saikosaponin b2	Triterpenoid saponin	CH	[15,30]

No.	formula	t_R, min	$[M+H]^+$ (error, ppm)	$[M-H]^-$ (error, ppm)	Major fragment ions in positive mode (m, z) b	Major fragment ions in negative mode (m, z) b	identification	Structure type	Source	Reference
					419.3278$[M+H-C_2H_2O-Glc-Fuc-3H_2O]^+$					
120	$C_{42}H_{68}O_{12}$	32.45	ND	763.4553（-4.6）	ND	809.4574$[M-H+HCOOH]^-$, 763.4553$[M-H]^-$, 601.4477$[M-H-C_6H_{10}O_5]^-$	Saikosaponin m	Triterpenoid saponin	CH	[20]
121	$C_{20}H_{18}O_6$	32.71	355.1162（-3.9）	353.1008（-6.4）	355.1161 299.0565$[M+H-C_4H_7]^+$	353.1004 285.1101$[M-H-C_5H_9]^-$, 163.0011$[M-H-C_5H_9-C_6H_4O_3]^-$,	Licoisoflavone A	Flavonoids	GC	[25]
122	$C_{21}H_{20}O_6$	32.84	369.1330（-0.6）	367.1166（-5.8）	369.1313 313.0691$[M+H-C_4H_7]^+$, 285.0744$[M+H-C_5H_9-CH_3]^+$, 271.0583$[M+H-C_5H_9-CH_3O]^+$ 243.0587	367.1175 309.0390$[M-H-C_3H_6-CH_3]^-$, 297.0398$[M-H-C_5H_9]^-$, 284.0287$[M-H-C_5H_9-CH_3]^-$	Glycycoumarin	Phenylpropanoids	GC	[6]
123	$C_{36}H_{58}O_8$	32.85	ND	617.4028（-6.9）	ND	663.4065$[M+COOH]^-$, 617.4246$[M-H]^-$	Prosaikogenin D	Triterpenoid saponin	CH	[6]
124	$C_{42}H_{68}O_{12}$	33.08	ND	763.4554（-3.8）	ND	809.4136$[M+COOH]^-$, 763.4554$[M-H]^-$, 601.4007$[M-H-C_6H_{10}O_5]^-$	Saikosaponin e	Triterpenoid saponin	CH	[25]
125	$C_{36}H_{58}O_8$	33.34	ND	617.3950（-3.8）	ND	663.4025$[M+COOH]^-$, 617.4454$[M-H]^-$	Prosaikogenin G	Triterpenoid saponin	CH	[25]

No.	formula	t_R, min	$[M+H]^+$ (error, ppm)	$[M-H]^-$ (error, ppm)	Major fragment ions in positive mode (m, z) [b]	Major fragment ions in negative mode (m, z) [b]	identification	Structure type	Source	Reference
126	$C_{20}H_{16}O_6$	33.48	353.1018 （-0.4）	351.0849 （-5.5）	353.1016<br>153.0175$[M+H-C_{13}H_{12}O_2]^+$	351.0847<br>321.0396$[M-H-2CH_3]^-$,<br>203.0367$[M-H-C_7H_4O_4]^-$,	Licoisoflavone B	Flavonoids	GC	[25]
127	$C_{44}H_{70}O_{14}$	33.66	823.4827 （-1.4）	821.4650 （-4.4）	823.4812$[M+H]^+$,<br>805.4756$[M+H-H_2O]^+$,<br>641.4089<br>455.3510$[M+H-C_2H_2O\text{-Glc-Fuc-}H_2O]^+$,<br>437.3392$[M+H-C_2H_2O\text{-Glc-Fuc-}2H_2O]^+$,<br>419.3276$[M+H-C_2H_2O\text{-Glc-Fuc-}3H_2O]^+$,	867.4648$[M+COOH]^-$,<br>821.1650$[M-H]^-$,	4″-O-Acetyl-Saikosaponin A	Triterpenoid saponin	CH	[6]
128	$C_{44}H_{70}O_{14}$	34.04	823.4815 （-2.9）	821.4648 （-4.6）	823.4805$[M+H]^+$,<br>805.4653$[M+H-H_2O]^+$,<br>787.4477<br>455.3505$[M+H-C_2H_2O\text{-Glc-Fuc-}H_2O]^+$,<br>437.3398$[M+H-C_2H_2O\text{-Glc-Fuc-}2H_2O]^+$,	867.4702$[M+COOH]^-$,<br>821.4648$[M-H]^-$,	4″-O-Acetyl Saikosaponin B2	Triterpenoid saponin	CH	[20]
129	$C_{46}H_{72}O_{15}$	34.61	865.4934 （-1.2）	863.4714 （-5.2）	ND	909.4688$[M+COOH]^-$,<br>863.4714$[M-H]^-$,<br>821.4593$[M-H-C_2H_2O]^-$,<br>761.4691$[M-H-2C_2H_2O-H_2O]^-$,<br>617.3882$[M-H-2C_2H_2O\text{-Glc}]^-$,	Diacetyl Saikosaponin A	Triterpenoid saponin	CH	[20]
130	$C_{20}H_{18}O_6$	34.89	355.1174 （-0.6）	353.1008 （-5.8）	355.1134<br>299.0543$[M+H-C_4H_7]^+$	353.1011<br>285.1110$[M-H-C_5H_9]^-$,<br>125.0236$[M-H-C_5H_9-C_9H_6O_3]^-$	Gancaonin L	Flavonoids	GC	[20]
131	$C_{20}H_{20}O_4$	36.08	ND	323.1273 （-4.6）	ND	323.1259$[M-H]^-$,<br>201.0920$[M-H-H_2O]^-$,<br>135.0451$[M-H-C_{12}H_{12}O_2]^-$,	Glabridin [c]	Flavonoids	GC	[6]

No.	formula	t_R, min	$[M+H]^+$ (error, ppm)	$[M-H]^-$ (error, ppm)	Major fragment ions in positive mode (m, z) [b]	Major fragment ions in negative mode (m, z) [b]	identification	Structure type	Source	Reference
132	$C_{21}H_{22}O_5$	37.92	355.1517 （-6.6）	353.1380 （-4）	355.1069<br>189.0923$[M+H-CH_3-C_8H_6O_3]^+$<br>153.0529$[M+H-C_{13}H_{14}O_2]^+$	353.1358<br>338.1126$[M-H-CH_3]^-$<br>150.0316$[M-H-CH_3-C_{12}H_{13}O_2]^-$	Licochalcone D	Flavonoids	GC	[20]
133	$C_{30}H_{46}O_4$	39.82	471.3454 （-3.1）	469.3278 （-8.5）	471.3442$[M+H]^+$,	469.3279$[M-H]^-$,<br>425.3379$[M-H-CO_2]^-$,	Glycyrrhetinic acid （enoxolone） [a, c]	Triterpenoid saponin	GC	[6]

Footnote:“a” Compared with reference standards; “b” The losses are: Glc= glucose moiety, Fuc= fructose；ND=not detect；“c” Confirmation in comparison with mass spectral library (Natural Products HR-MS, MS Spectral Library, Version 1.0; AB Sciex, Foster City, USA).

CH：Bupleuri Radix，HQ：Scutellariae radix，SJ：Zingiberis rhizoma recens，JBX：Pinelliae rhizoma praeparatum cum zingibere et alumine，DZ：Jujubae fructus，DS：Codonopsis radix，GC：Glycyrrhizae Radix.

Table S3 Results of calibration curve and content for the determination of 9 compounds

**Name**	**Regression equation**	**R^2**	**Linear range (μg/ml)**	**Contents (mg/g)**
baicalin	y = 6.7581x + 0.4186	0.9961	0.53~4.74	3.25
baicalein	y = 4.8933x + 9.445	0.9999	1.24~11.20	0.34
Wogonin	y = 6.8079x + 0.4146	0.9983	6.32~61.97	0.2
Wogonoside	y = 5.5312 x + 3.6203	0.9990	9.77~87.89	2.8
quercetin	y = 6.3909x + 0.4414	0.9991	0.08~0.72	0.01
Saikosaponin B1	y = 3.8802x + 0.4141	0.9994	0.86~7.73	0.2
Saikosaponin B2	y = 3.854x + 0.4052	0.9998	0.89~7.97	0.25
glycyrrhizic acid	y = 5.0736x + 8.023	0.9974	1.15~10.34	2.05
Liquiritin	y = 4.8287x + 0.9097	0.9955	1.10~9.82	0.54

Table S4 The active ingredients of XCHG

Name	chemical compound
X1	Glycyrrhetinic Acid
X2	5,7,8-Trihydroxy-6-methoxyflavone
X3	Aspartic acid
X4	Baicalein
X5	Betulonic acid
X6	Curcumin
X7	Desaminotyrosine
X8	Formononetin
X9	Glabridin
X10	Glycyrrhizic acid
X11	Isoliquiritigenin
X12	Licoisoflavone A
X13	Licoisoflavone B
X14	Licoricesaponin G2
X15	liquiritigenin
X16	Liquiritin
X17	Proline
X18	Quercetin
X19	Saikosaponin A
X20	Saikosaponin B1
X21	Saikosaponin B2
X22	Saikosaponin C
X23	Valine
X24	Wogonin
X25	Wogonoside
X26	Dihydrobaicalein
X27	Baicalin
X28	Neobaicalein
X29	Adenosine
X30	Lobetyolin

Table S5 Enriched KEGG signaling pathway/GO biological process of XCHG-fever targets

**Biological functional modules**	**Type**	**GO biological process/ KEGG signaling pathway**	***p*-value**
Inflammation/Immune	KEGG	NF-kappa B signaling pathway	9.72E-10
	KEGG	TNF signaling pathway	2.55E-15
	GO	inflammatory response	1.38E-16
Neuromodulation	KEGG	Neuroactive ligand-receptor interaction	1.94E-04
	KEGG	Serotonergic synapse	1.98E-07
	KEGG	Inflammatory mediator regulation of TRP channels	8.70E-05
	GO	calcium-mediated signaling	1.17E-05
Vasodilatory	KEGG	Relaxin signaling pathway	1.79E-10
	GO	positive regulation of nitric oxide biosynthetic process	1.73E-13
	GO	response to muscle stretch	3.97E-07
Metabolism	KEGG	Endocrine resistance	2.13E-20
	KEGG	cAMP signaling pathway	2.22E-08
	GO	glucose metabolic process	6.51E-06
	GO	positive regulation of cholesterol efflux	4.98E-05
	GO	positive regulation of prostaglandin secretion	7.06E-04

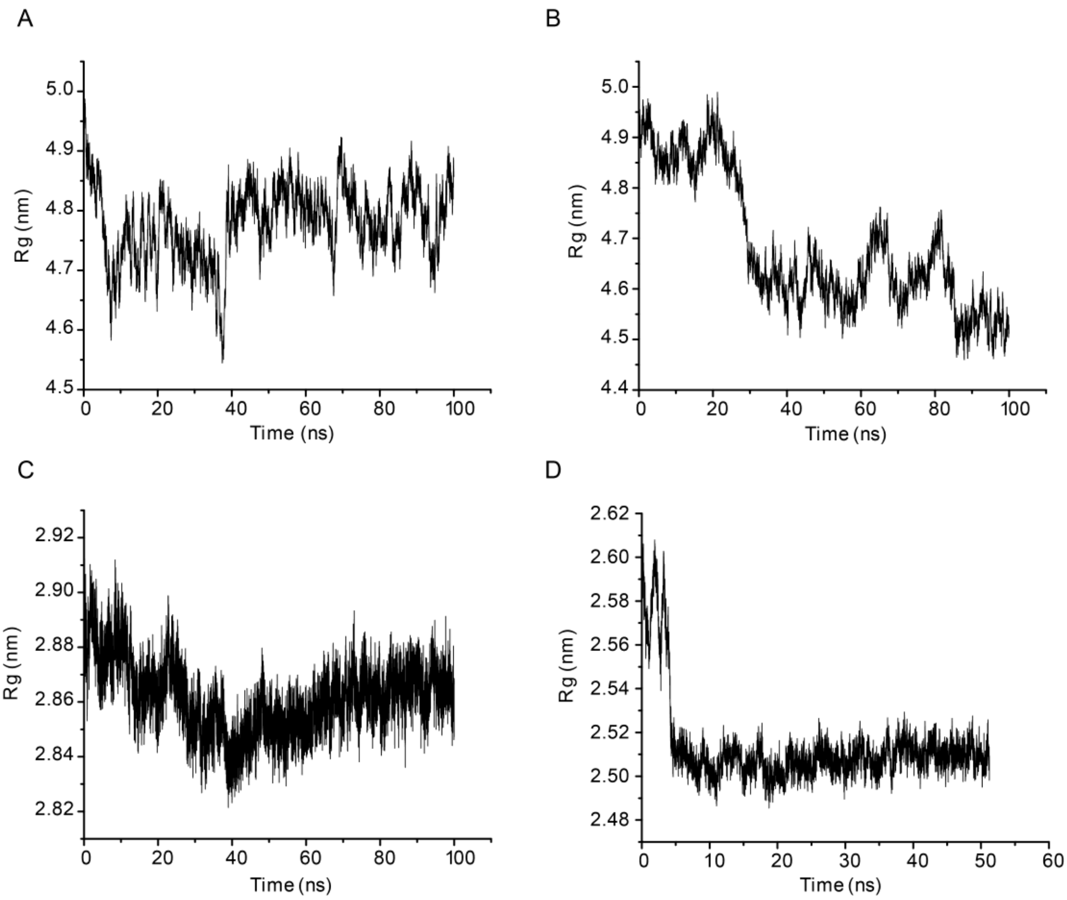

Figure S1. Radius of gyration (Rg) values of indicated complexes: GABBR2-saikosaponin C (**A**), GABBR2-baicalin (**B**), NFKBIA-glycyrrhizicacid (**C**) and PTGS2-lobetyolin (**D**) from the data of 100 ns MDs trajectories.

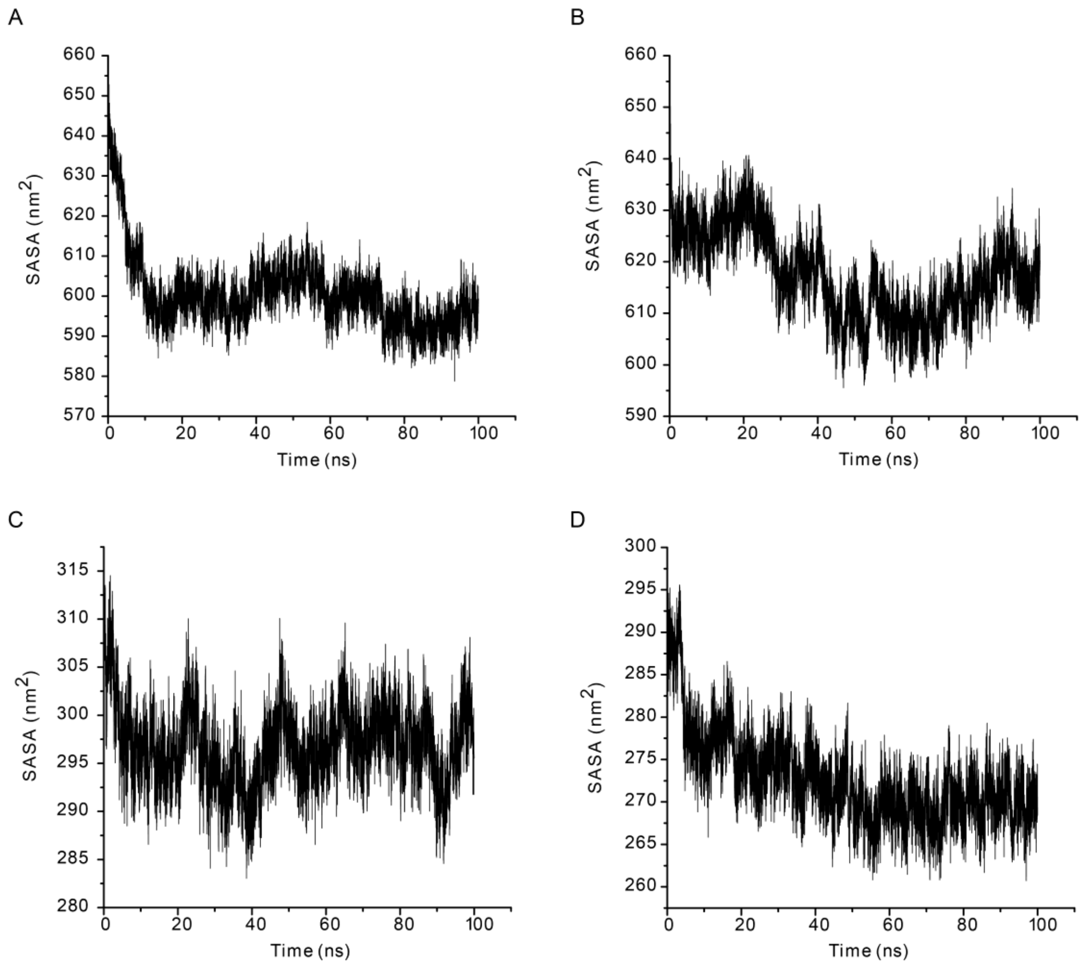

Figure S2. Solvent accessible surface area (SASA) values of indicated complexes: GABBR2-saikosaponin C (**A**), GABBR2-baicalin (**B**), NFKBIA-glycyrrhizicacid (**C**) and PTGS2-lobetyolin (**D**) from the data of 100 ns MDs trajectories.

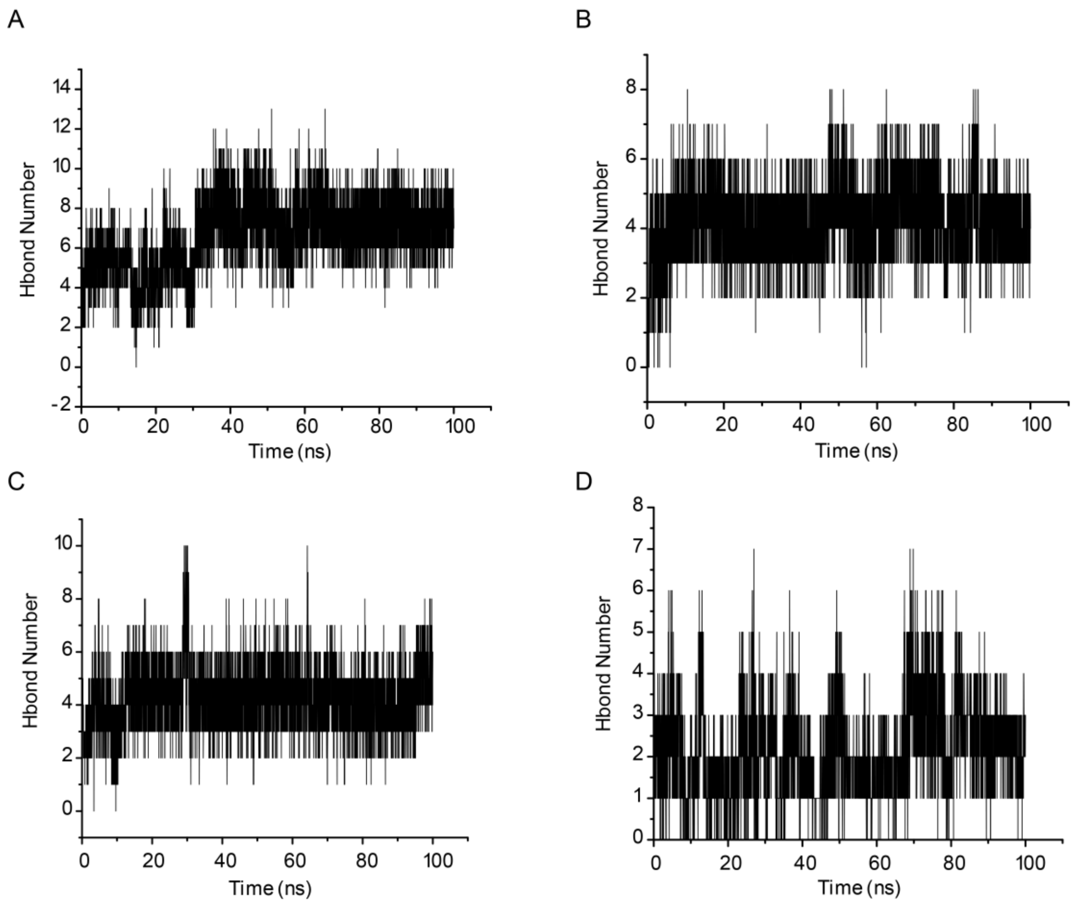

Figure S3. Hydrogen bond (Hbond) numbers of indicated complexes: GABBR2-saikosaponin C (**A**), GABBR2-baicalin (**B**), NFKBIA-glycyrrhizicacid (**C**) and PTGS2-lobetyolin (**D**) from the data of 100 ns MDs trajectories.

Reference

1. Wu, Y.; Peng, Y.; Song, C.; Li, L.; Ma, H.; Li, D.; Wang, F.; Yang, J.; Song, S.; Wu, C. Separation and identification of multiple constituents in Xiao Chai Hu Decoction (Sho-saiko-to) by bioactivity-guided fractionation combined with LC-ESI-QTOFMS/MS. *Biomed Chromatogr* **2015**, *29*, 1146-1166. https://doi.org/10.1002/bmc.3402.
2. Guo, S.; Duan, J.A.; Qian, D.; Tang, Y.; Qian, Y.; Wu, D.; Su, S.; Shang, E. Rapid determination of amino acids in fruits of Ziziphus jujuba by hydrophilic interaction ultra-high-performance liquid chromatography coupled with triple-quadrupole mass spectrometry. *J Agric Food Chem* **2013**, *61*, 2709-2719. https://doi.org/10.1021/jf305497r.
3. Gao, S.; Liu, J.; Wang, M.; Liu, Y.; Meng, X.; Zhang, T.; Qi, Y.; Zhang, B.; Liu, H.; Sun, X.; et al. Exploring on the bioactive markers of Codonopsis Radix by correlation analysis between chemical constituents and pharmacological effects. *Journal of Ethnopharmacology* **2019**, *236*, 31-41. https://doi.org/10.1016/j.jep.2019.02.032.
4. Ma, X.Q.; Leung, A.K.; Chan, C.L.; Su, T.; Li, W.D.; Li, S.M.; Fong, D.W.; Yu, Z.L. UHPLC UHD Q-TOF MS/MS analysis of the impact of sulfur fumigation on the chemical profile of Codonopsis Radix (Dangshen). *Analyst* **2014**, *139*, 505-516. https://doi.org/10.1039/c3an01561k.
5. Chen, T.; Wang, X.; Chen, P.; Zheng, Y.; He, Y.; Zeng, X.; Peng, W.; Su, W. Chemical components analysis and in vivo metabolite profiling of Jian'er Xiaoshi oral liquid by UHPLC-Q-TOF-MS/MS. *Journal of Pharmaceutical and Biomedical Analysis* **2022**, *211*, 114629. https://doi.org/10.1016/j.jpba.2022.114629.
6. Tan, G.; Zhu, Z.; Zhang, H.; Zhao, L.; Liu, Y.; Dong, X.; Lou, Z.; Zhang, G.; Chai, Y. Analysis of phenolic and triterpenoid compounds in licorice and rat plasma by high-performance liquid chromatography diode-array detection, time-of-flight mass spectrometry and quadrupole ion trap mass spectrometry. *Rapid Commun. Mass Spectrom.* **2010**, *24*, 209-218. https://doi.org/10.1002/rcm.4373.
7. Han, J.; Ye, M.; Xu, M.; Sun, J.; Wang, B.; Guo, D. Characterization of flavonoids in the traditional Chinese herbal medicine-Huangqin by liquid chromatography coupled with electrospray ionization mass spectrometry. *Journal of Chromatography B* **2007**, *848*, 355-362. https://doi.org/10.1016/j.jchromb.2006.10.061.
8. Farag, M.A.; Porzel, A.; Wessjohann, L.A. Comparative metabolite profiling and fingerprinting of medicinal licorice roots using a multiplex approach of GC–MS, LC–MS and 1D NMR techniques. *Phytochemistry* **2012**, *76*, 60-72. https://doi.org/10.1016/j.phytochem.2011.12.010.
9. Liao, W.C.; Lin, Y.-H.; Chang, T.-M.; Huang, W.-Y. Identification of two licorice species, Glycyrrhiza uralensis and Glycyrrhiza glabra, based on separation and identification of their bioactive components. *Food Chemistry* **2012**, *132*, 2188-2193. https://doi.org/10.1016/j.foodchem.2011.12.051.
10. Zhang, Q.; Ye, M. Chemical analysis of the Chinese herbal medicine Gan-Cao (licorice). *J Chromatogr A* **2009**, *1216*, 1954-1969. https://doi.org/10.1016/j.chroma.2008.07.072.
11. Yin, Q.; Wang, P.; Zhang, A.; Sun, H.; Wu, X.; Wang, X. Ultra-performance LC-ESI/quadrupole-TOF MS for rapid analysis of chemical constituents of Shaoyao-Gancao decoction. *Journal of Separation Science* **2013**, *36*, 1238-1246. https://doi.org/10.1002/jssc.201201198.
12. Shang, Z.; Liu, C.; Qiao, X.; Ye, M. Chemical analysis of the Chinese herbal medicine licorice (Gan-Cao): An update review. *J Ethnopharmacol* **2022**, *299*, 115686. https://doi.org/10.1016/j.jep.2022.115686.
13. Du, T.; Zeng, M.; Chen, L.; Cao, Z.; Cai, H.; Yang, G. Chemical and Absorption Signatures of Xiao Chai Hu Tang. *Rapid Commun Mass Spectrom* **2018**. https://doi.org/10.1002/rcm.8114.
14. Seo, O.N.; Kim, G.-S.; Kim, Y.-H.; Park, S.; Jeong, S.W.; Lee, S.J.; Jin, J.S.; Shin, S.C. Determination of polyphenol components of Korean Scutellaria baicalensis Georgi using liquid chromatography–tandem mass spectrometry: Contribution to overall antioxidant activity. *Journal of Functional Foods* **2013**, *5*, 1741-1750. https://doi.org/10.1016/j.jff.2013.07.020.

15. Zhang, F.; Li, Z.; Li, M.; Yuan, Y.; Cui, S.; Chen, J.; Li, R. An integrated strategy for profiling the chemical components of Scutellariae Radix and their exogenous substances in rats by ultra-high-performance liquid chromatography/quadrupole time-of-flight mass spectrometry. *Rapid Communications in Mass Spectrometry* **2020**, *34*, e8823. https://doi.org/10.1002/rcm.8823.
16. Qiao, X.; Li, R.; Song, W.; Miao, W.-j.; Liu, J.; Chen, H.-b.; Guo, D.-a.; Ye, M. A targeted strategy to analyze untargeted mass spectral data: Rapid chemical profiling of Scutellaria baicalensis using ultra-high performance liquid chromatography coupled with hybrid quadrupole orbitrap mass spectrometry and key ion filtering. *Journal of Chromatography A* **2016**, *1441*, 83-95. https://doi.org/10.1016/j.chroma.2016.02.079.
17. Montero, L.; Ibáñez, E.; Russo, M.; di Sanzo, R.; Rastrelli, L.; Piccinelli, A.L.; Celano, R.; Cifuentes, A.; Herrero, M. Metabolite profiling of licorice (Glycyrrhiza glabra) from different locations using comprehensive two-dimensional liquid chromatography coupled to diode array and tandem mass spectrometry detection. *Anal Chim Acta* **2016**, *913*, 145-159. https://doi.org/10.1016/j.aca.2016.01.040.
18. Zhou, Y.; Wang, M.-K.; Liao, X.; Zhu, X.-M.; Peng, S.-L.; Ding, L.-S. Rapid identification of compounds in Glycyrrhiza uralensis by liquid chromatography/tandem mass spectrometry. *Chinese journal of analytical chemistry* **2004**, *32*, 174-178, https://api.semanticscholar.org/CorpusID:100157464.
19. Morimoto, S.; Harioka, T.; Shoyama, Y. Purification and characterization of flavone-specific β-glucuronidase from callus cultures of Scutellaria baicalensis Georgi. *Planta* **1995**, *195*, 535-540. https://doi.org/10.1007/BF00195712.
20. Lei, T.; Chen, S.; Wang, K.; Zhang, D.; Dong, L.; Lv, C.; Wang, J.; Lu, J. Characterization and discrimination of raw and vinegar-baked Bupleuri radix based on UHPLC-Q-TOF-MS coupled with multivariate statistical analysis. *Biomedical Chromatography* **2018**, *32*, e4044. https://doi.org/10.1002/bmc.4044.
21. Xuan, C.; Zhao, X.; Feng, F.; Qiao, B. The study of chemical components of Glycyrrhiza in Shanxi. *Natural Product Research and Development* **2000**, *12*, 18-22, https://europepmc.org/article/CBA/333984.
22. Miyaichi, Y.; Imoto, Y.; Tomimori, T.; Namba, T. Studies on the Nepalese crude drugs. IX. On the flavonoid constituents of the root of Scutellaria scandens Buch.-Ham. ex D. Don. *Chem. Pharm. Bull.* **1988**, *36*, 2371. https://doi.org/10.1248/cpb.36.2371.
23. Liu, G.; Rajesh, N.; Wang, X.; Zhang, M.; Wu, Q.; Li, S.; Chen, B.; Yao, S. Identification of flavonoids in the stems and leaves of Scutellaria baicalensis Georgi. *Journal of Chromatography B* **2011**, *879*, 1023-1028. https://doi.org/10.1016/j.jchromb.2011.02.050.
24. Liu, G.; Ma, J.; Chen, Y.; Tian, Q.; Shen, Y.; Wang, X.; chen, B.; Yao, S. Investigation of flavonoid profile of Scutellaria bacalensis Georgi by high performance liquid chromatography with diode array detection and electrospray ion trap mass spectrometry. *Journal of Chromatography A* **2009**, *1216*, 4809-4814. https://doi.org/10.1016/j.chroma.2009.04.021.
25. Shan, L.; Yang, N.; Zhao, Y.; Sheng, X.; Yang, S.; Li, Y. A rapid classification and identification method applied to the analysis of glycosides in Bupleuri radix and liquorice by ultra high performance liquid chromatography coupled with quadrupole time-of-flight mass spectrometry. *Journal of Separation Science* **2018**, *41*, 3791-3805. https://doi.org/10.1002/jssc.201800619.
26. Bai, M.; Zheng, C.-J.; Wu, L.-J.; Wu, S.-Y.; Cai, Y.; Chen, G.-Y.; Han, C.-R.; Song, X.-P. Bioactive flavonoid derivatives from Scutellaria luzonica. *Chemistry of Natural Compounds* **2018**, *54*, 350-353. https://doi.org/10.1007/s10600-018-2342-y.
27. Ren, Q.; Xia, T.; Quan, X.-G.; Ding, L.; Wang, H.-Y. Antileukemic activity of the chemical constituents from scutellaria barbata d. don. *Acta Chromatogr.* **2017**, *29*, 399-413. https://doi.org/10.1556/1326.2017.29.3.10.
28. Lee, S.M.; Min, B.S.; Lee, C.G.; Kim, K.S.; Kho, Y.H. Cytotoxic triterpenoids from the fruits of Zizyphus jujuba. *Planta Med* **2003**, *69*, 1051-1054. https://doi.org/10.1055/s-2003-45155.
29. Lee, S.M.; Park, J.G.; Lee, Y.H.; Lee, C.G.; Min, B.S.; Kim, J.H.; Lee, H.K. Anti-

complementary activity of triterpenoides from fruits of Zizyphus jujuba. *Biol Pharm Bull* **2004**, *27*, 1883-1886. https://doi.org/10.1248/bpb.27.1883.

30. Jiang, Y.; Liu, R.; Chen, J.; Liu, M.; Liu, M.; Liu, B.; Yi, L.; Liu, S. Application of multifold characteristic ion filtering combined with statistical analysis for comprehensive profiling of chemical constituents in anti-renal interstitial fibrosis I decoction by ultra-high performance liquid chromatography coupled with hybrid quadrupole-orbitrap high resolution mass spectrometry. *Journal of Chromatography A* **2019**, *1600*, 197-208. https://doi.org/10.1016/j.chroma.2019.04.051.
